# The efficacy of bone marrow-derived mesenchymal stem cells in restoring limbal stem cell deficiency in rat model

**DOI:** 10.1038/s41598-025-26637-2

**Published:** 2025-11-20

**Authors:** Mohamed A. El-Desouky, Nadia Samy Mahmoud, Fayek M. Ghaleb, Fatma B. Rashidi, Iman M. A. Zaki, Marwa A. Fouly, Ahmed M. Ata, Hanaa Hamdy Ahmed

**Affiliations:** 1 Biochemistry Division, Chemistry Department, Faculty of Science, Cairo University, Giza, Egypt; 2 Hormones Department, Medical Research and Clinical Studies Institute, National Research Centre, Dokki, Giza, Egypt; 3 Stem Cells Lab, Center of Excellence for Advanced Sciences, National Research Centre, Dokki, Giza, Egypt; 4 Clinical Pathology Department, Research Institute of Ophthalmology, Giza, Egypt; 5 Pathology Department, Research Institute of Ophthalmology, Giza, Egypt; 6 Ophthalmology Department, Research Institute of Ophthalmology, Giza, Egypt; 7 Biochemistry Department, Research Institute of Ophthalmology, Giza, Egypt

**Keywords:** Limbal stem cell deficiency, BM-MSCs, Alkali burn, Inflammation, Angiogenesis, Apoptosis, Cell biology, Diseases, Medical research, Stem cells

## Abstract

This approach aimed to assess the therapeutic significance of BM-MSCs in alleviating LSCD elicited in rats *via* corneal alkali burns and to explore the associated repairing mechanisms. Rats were assigned to (1) control group, (2) alkali burn group, and (3) stem cells group. Corneal surface investigations were performed using a slit lamp and fluorescence tests. The therapeutic impact of BM-MSCs was appraised by estimating the protein levels of TNF-α, SOD, VEGF, MMP-2, and IL-8 using ELISA. Moreover, mRNA transcription levels of corneal TNF-α, NF-kB, COX-2, and caspase-3 were quantified. Histological investigation and immunostaining of corneal cytokeratin 3 and p63 proteins were also conducted. The results indicated that BM-MSCs treatment enhanced the corneal wound healing, decreased corneal opacity, and neovascularization. Moreover, such treatment resulted in a significant decline in corneal TNF-α, IL-8, VEGF, and MMP-2 protein levels. Additionally, stem cells group elicited a significant down-expression of TNF-α, NF-κB, COX-2, and caspase-3 genes. Besides, BM-MSCs treatment restored the corneal epithelial integrity and ameliorated the pathological characteristics, as confirmed by histological and immunohistochemical analysis. Conclusively, the present investigation underscores the healing ability of BM-MSCs in LSCD in the rat model by repairing the damaged corneal epithelium through withstanding inflammation, angiogenesis, and apoptosis. Consequently, MSCs transplantation represents a promising therapeutic modality for LSCD.

**Supplementary Information:**

The online version contains supplementary material available at 10.1038/s41598-025-26637-2.

## Introduction

LSCD is an eye disorder, recognized by the lack of stem cells in the limbus, which has a vital role in corneal epithelium re-population and maintains the barrier function of the limbus. This leads to partial or complete loss of the regenerative ability of the cornea, resulting in corneal scarring, loss of normal corneal luster, and invasion of the conjunctival cells to the cornea “conjunctivalization”, leading to corneal opacification, chronic inflammation, neovascularization, and eventually to complete blindness^1^.

LSCD originates from primary factors, including aniridia^2^, congenital epidermal dysplasia^3^, and Turner syndrome^4^. Moreover, it can be caused by secondary factors, including external stimuli that damage the stem cell niche, destroy LSCs, or both, such as chemical injuries and chronic inflammation^5^, Stevens-Johnson syndrome^1^, injury caused by surgeries in the limbic region, or drug toxicity^3^.

LSCD patients suffer from chronic conjunctival redness, poor epithelial wound healing, foreign body sensation, photophobia, tearing, and decreased vision^6^. Many treatment options have been described for LSCD management, including medical management^7^ and surgical interventions^8^. However, no appropriate curative strategy for LSCD has been identified yet. Since medical treatments including anti-inflammatory and growth factor medications are not effective in the advanced stages of LSCD, therefore LSCD patients seek corneal surgeries despite their side effects, including; iatrogenic LSCD in the eye of donor in case of autologous procedure in addition to the risk for graft rejection in case of bilateral LSCD that is based on allogeneic limbal grafts, so patients have to undergo long-term systemic immunosuppression drug course^9^. Hence, further studies are required to find an appropriate treatment for LSCD.

Mesenchymal stem cells (MSCs) are multipotent stromal cells. They are extracted from adipose tissue, bone marrow, peripheral blood, and Wharton’s jelly of umbilical cord blood. MSCs retain specific properties, including plastic adherence; acquiring particular cell surface antigens, including CD105, CD90, and CD73; and the absence of hematopoietic and endothelial markers (CD11b, CD45, CD14, CD34, CD19, and HLA-DR). Moreover, it can differentiate into chondrocyte, osteoblast, and adipocyte lineages in vitro^10^.

MSCs have been utilized as a treatment for regenerating damaged cells, as they can facilitate the plasticity of damaged tissues and release neurotrophic and survival-promoting growth factors^11^. They also possess powerful immunomodulatory characteristics and hinder the liberation of pro-inflammatory mediators^12^.

Some studies employing MSCs for treating LSCD and corneal wounds reported promising results^13–15^. However, further studies are necessary to elucidate the biochemical and molecular mechanisms elicited by MSCs in treating LSCD to establish its therapeutic impact.

Therefore, this work was designed to explore the potential therapeutic impact of bone marrow-derived MSCs in counteracting the murine alkali burn-induced LSCD by investigating the influence of such treatment in mitigating the inflammatory response and angiogenesis associated with LSCD, *via* biochemical, molecular, and histological investigations.

## Materials and methods

### Harvesting and culture of BM-MSCs

Bone marrow cells were obtained by flushing the bone marrow cavity of femur and tibia bones of eight-week-old female *Wistar* rats (100–130 g) using a sterile syringe containing high glucose Dulbecco’s modified Eagle’s medium (HG-DMEM, Biowest, France) provided with 10% heat-inactivated fetal bovine serum (Biowest, France) and 1% penicillin/streptomycin (Biowest, France). The obtained cells were centrifuged, and the cell pellets were then seeded into a 25 cm^2^ tissue culture flask containing a basal medium and incubated at 37 °C in a 5% humidified CO_2_ incubator (Sartorius Stadium Biotech, Germany). Non-attached cells were discarded after 2 days with PBS, and the adherent cells were replenished with a fresh complete medium. The media were replaced every 3 days until MSCs cultures reached 80–90% confluent cell sheet. Cell passaging was performed until obtaining the 3rd passage cultures^16^.

### Verification of mesenchymal stem cell characteristics

BM-MSC cultures were observed using the inverted microscope (Olympus, Japan) to detect their growth and morphological characteristics. MSCs-specific markers (CD105 and CD90) and hematopoietic stem cell marker (CD34) were identified using flow cytometry analysis to ensure the identity of the MSCs^17^. Briefly, the third passage culture of BM-MSCs was immersed in PBS supplied with 0.5% bovine serum albumin (BSA)/ EDTA (2 mmol). Then, the cells were screened for surface antigen manifestation using anti-rat phycoerythrin (PE)-conjugated CD90 antibody (BD Bioscience, USA), anti-rat PE-labeled CD105 antibody (Thermofisher, USA), and anti-rat fluorescein isothiocyanate FITC-conjugated CD34 antibody (Thermofisher, USA). After that, the cells were rinsed two times with PBS supplemented with BSA (2%), replenished in PBS, and evaluated using flow cytometry (Beckman Coulter Elite XL, USA instrument). Isotype control rat immunoglobulin was utilized to detect background staining.

### In vivo experiment

#### Animals

Sixty-six mature female *Wistar* rats with a weight range from 200 to 220 g were acquired from the animal house colony of the Research Institute of Ophthalmology, Giza, Egypt. The rats were accommodated individually in a Ventilated Caging System at a temperature of 25 °C, and supplied with tap water and rodent chow. Anesthesia of all rats was carried out by intraperitoneal injection of 65 mg/kg Ketamine (purchased from Sigmatec, Egypt) and 10 mg/kg Xylazine (purchased from ADWIA, Egypt), in addition to receiving local anesthesia with Benox ophthalmic drops (purchased from EIPICO, Egypt).

Ethical approval statement: All experimental protocols of the current study were approved by the Institutional Animal Care and Use Committee (IACUC) of Cairo University, Giza, Egypt (**Approval No. CU-I-F-21-20**), and were conducted in compliance with the ARRIVE guidelines.

#### Limbal stem cell deficiency induction

After complete anesthesia of rats, LSCD was induced by corneal alkali burn *via* placing a filter paper ring (4 mm in diameter) saturated with 0.5 mol/L sodium hydroxide (AnalaR) on the whole corneal surface, including the limbal region, of the right eyes of the rats for 20 s, followed by rinsing with 0.9% saline for 1 min. To prevent any contamination of the corneal surface, Isopto Maxitrol antibiotic drops, purchased from Novartis Co., Belgium, were applied to the injured eyes three times a day till the end of the experiment, according to the modified methods of Jiang et al.^14^ and Yao et al.^15^.

### Experimental protocol

All animals were left to adapt to the surrounding conditions for one week before initiating the experiments. After that, rats were equally classified into three groups (22 rats/ group); **Group (1)** represented the control rats that received a subconjunctival injection with 0.1 ml phosphate buffer saline (PBS) in the right eyes following general anesthesia, **Group (2)**: the alkali burn group; in which rats were subjected to corneal alkali injury in the right eyes for induction of limbal stem cell deficiency and undergone a subconjunctival injection of 0.1 ml PBS, instantly and after 2 days of corneal alkali burn, and **Group (3)**: Stem cells group represented corneal alkali burn-induced rats received a subconjunctival injection in the right eyes with two doses (2 × 10^6^ cells/rat eye) of BM-MSCs suspended in 0.1 ml PBS immediately and after 2 days of corneal alkali burn, respectively^15^. Rats were sacrificed at the end of the experiment, after 7 days from the induction day.

### Follow-up and ocular surface examination

Rats were subjected to ocular surface examination using a slit lamp (Zeiss HSO-10 portable slit lamp 6 V 10 W OSRAM 64222 with focal length 125) to determine the scores for opacity, neovascularization grade, and ulcer development. Rats’ corneas were photographed and subjected to topical fluorescence staining using fluorescein strips at day zero (6 h following alkali burn and stem cell injection), day 2, and day 7. The corneal examinations were blindly performed. A smartphone camera was used to capture all photographs from the eyepiece of the portable slit lamp device.

Corneal opacity was determined by grades according to its density as follows; grade 0 refers to a clear cornea, grade 1 represents the least density observed with difficulty, grade 2 indicates minor haze that could be easily detected, grade 3 refers to moderately dense opacity that partly blocks the visibility of iris details and grade 4 indicates extremely dense opacity that entirely blocks the visibility of the intraocular structure details. The size of the corneal opacity patch was measured by using Image J 1.54d software. Corneal neovascularization was scored in grades according to the amount and the extent of newly formed vessels measured using Image J 1.54d software, as follows; grade 0 indicates no intracorneal vessels and normal corneoscleral limbus, grade 1 indicates that smaller than five vascular loops were observed in the corneal surface with length ≤ 0.3 mm, grade 2: in which about (5–15) vascular loops observed in the corneal surface with a length ≤ 0.3 mm, grade 3: greater than 15 vessels or loops observed in the corneal surface with length > 0.3 mm, and finally grade 4: ≥ 2 loops observed with length > 0.5 mm^13^.

### Tissue sampling

All rats were sacrificed under general anesthesia, as mentioned before, and their eyes were enucleated and kept in ice-cold saline. Four eyes from each group were fixed in 4% paraformaldehyde for histological and immunohistochemical examinations. The remaining eyes have been dissected, the corneas isolated, and frozen at -80 °C for further biochemical determinations, molecular genetics assessment, and protein immunoblotting analysis.

### Specimen preparation for biochemical determination

Rat corneas were dissected from the eyes and then rinsed in ice-cold PBS (pH 7.4). Cornea tissues were minced and homogenized in ice-cold PBS at a ratio (0.2 g tissue: 1 ml buffer) using a glass rod. Pooled samples were taken from each five corneal tissues and centrifuged at 14,000 rpm for 20 min. using a cooling centrifuge (Tehtnica, Centric 200R, Slovenia). The resultant supernatants of 6 pooled samples were used for biochemical analysis.

### Biochemical determinations

All ELISA kits were purchased from Bioassay Technology Laboratory Company (China) for estimation of the following biochemical parameters in cornea tissue homogenates: TNF-α (Cat.no. E0764Ra), VEGF (Cat.no. E0659Ra), SOD (Cat.no. E0164Rb), MMP-2 (Cat.no. E0315Ra), and IL-8 (Cat.no. E0044Rb). All the obtained data were calculated relative to the level of total protein estimated in corneal tissue homogenates by the Bradford method using the Protein Assay Kit (Cat.no. MBS355526) purchased from MyBioSource Company - USA.

### Gene expression assessment by Real-time PCR

The mRNA expression levels of TNF-α, NF-κB, COX-2, and Caspase-3 were evaluated by quantitative polymerase chain reaction (qRT-PCR) using the DNA-Technology Real-Time PCR device (DTlite 4, Russia). RNeasy mini kit (Cat. #74104, Qiagen, Germany) was utilized to isolate the RNA from the corneal tissue of the rats. RNA quantity and integrity were estimated by NanoDrop 2000 (Thermo Fisher Scientific, Rockford, IL, USA) using a 260/280 nm ratio. RevertAid cDNA synthesis kit (Cat# K1621, Thermo Fisher Scientific, Lithuania) was used for the reverse transcription of the extracted RNA into cDNA according to the provided protocol. PCR mixture included 10 µM of each sense and anti-sense primers of the target genes (Invitrogen, USA), 12.5 µl Maxima SYBR Green qPCR master mix (Thermo Fisher Scientific), 100 ng of cDNA, and nuclease-free water. Table 1 entails the primer pairs of target genes. The amplification program included one step of initial denaturation at 95 °C for 10 min, followed by 40 repetitive cycles of 15 s denaturation at 95 °C, 30 s annealing at 60 °C, and 30 s extension at 72 °C. The 2^-ΔΔCt^ method was used to evaluate the comparative mRNA transcriptional levels relative to the control value. GAPDH was utilized as an internal control.

**Table 1:** Primer pairs of corneal ulcer-related genes used in qRT-PCR.

Genes	Primer sequences	References
NF-κB	F:5’-GCACGGATGACAGAGGCGTGTATAAGG-3’	^18^
	R: 5’- GGCGGATGATCTCCTTCTCTCTGTCTG − 3’	
TNF-α	F: 5’-CAGACCCTCACACTCAGATCATCTT-3’	^19^
	R: 5’-CAGAGCAATGACTCCAAAGTAGACCT-3’	
GAPDH	F: 5’-CACCCTGTTGCTGTAGCCATATTC-3’	^20^
	R: 5’-GACATCAAGAAGGTGGTGAAGCAG-3’	
COX-2	F: 5’-CTCCTTGAACACGGACTTGC-3’	^21^
	R: 5’-TCAGGGAGAAGCGTTTGC-3’	
Caspase-3	F: 5’-TGGTACCGATGTCGATGCAGC-3’	^22^
	R: 5’-GGTCCACAGGTCCGTTCGTT-3’	
	R: 5’-TAGGTGAGGGCTTGCCTGAGTG-3’	

### Protein immunoblotting analysis of NF-kB

The expression level of NF-kB protein in the corneal tissue was evaluated using a Western blot. Corneal tissues were lysed in ice-cold RIPA lysis buffer to obtain homogenates. Then, the tissue lysates were separated by centrifugation at 12,000 rpm for 20 min at 4 °C. The total protein concentration of the supernatants was detected by the Bradford method. Protein samples (25 µg) were separated by 10% SDS polyacrylamide gel electrophoresis under a voltage of 200 V and then electro-transferred onto PVDF membrane (0.2 μm SIGMA) using mini–Trans-Blot Module (Bio-Rad, USA). The membrane was blocked by immersing in Tris-buffered saline/0.1% Tween-20 (TBST) supplied with 5% skimmed milk for 1 h. Then, it was incubated with the anti-NF-kB antibody purchased from FAGUS, UK (Cat no. SKU FAS-22066-A, 1:1000 dilution in TBST supplied with 5% Bovine Serum Albumin) overnight at 4 °C. After washing the membrane with TBST, it was incubated with rabbit anti-mouse IgG polyclonal antibody horseradish peroxidase (Cat no. 31450, Invitrogen, 1:3000 dilution in TBST supplied with 5% skimmed milk) for 60 min at room temperature. Ultimately, the protein bands were visualized on the PVDF membrane with the enhanced chemiluminescent substrate (Cat. No. 32106, Thermo Scientific, USA) following the manufacturer’s recommendation, after washing the membrane with TBST.

### Histological examination

After the cornea tissues were fixed in 4% paraformaldehyde overnight, they were cut, processed, embedded in paraffin wax blocks, and sectioned using a microtome (Leica, USA) at 4 μm thickness. Staining of corneal tissue sections with H&E stain was performed for light microscopic observation.

### Immunohistochemical investigation

Immunostaining of Cytokeratin 3 and p63 proteins was detected in the paraffin-embedded corneal epithelial tissue sections according to the avidin-biotin-peroxidase complex (ABC) method^23^. The primary antibodies used were the Rabbit p63 Monoclonal Antibody (Novus biological, Cat. No. NBP3-32703) diluted at a ratio 1:200, and the mouse Cytokeratin 3 Monoclonal Antibody (Novus Biologicals, Cat. No. NBP2-79718) diluted at a ratio 1:100. The tissue sections of different groups were incubated with each primary antibody, followed by treatment with the reagents of the Vectastain ABC-HRP kit (Vector Laboratories, Newark, CA). The antigen–antibody reactions were visualized using avidin-biotin peroxidase complex and diaminobenzidine (DAB) (Sigma). The negative control was prepared by replacing the primary antibody with non-immune serum. The immunostained slides were then investigated using a light microscope (Olympus BX-53, Japan). The quantification of the staining intensity was performed using ImageJ software (version 1.54 J), after being normalized to the tissue area in each slide.

### Statistical analysis

The current data were analyzed using SPSS (Version 19) software. All data were represented as mean ± S.D. The significance between groups was assessed using one-way ANOVA, followed by the LSD test. The mean difference was recognized to be significant when *p <* 0.05^24^.

## Results

### BM-MSCs identification

**Fig. 1:**
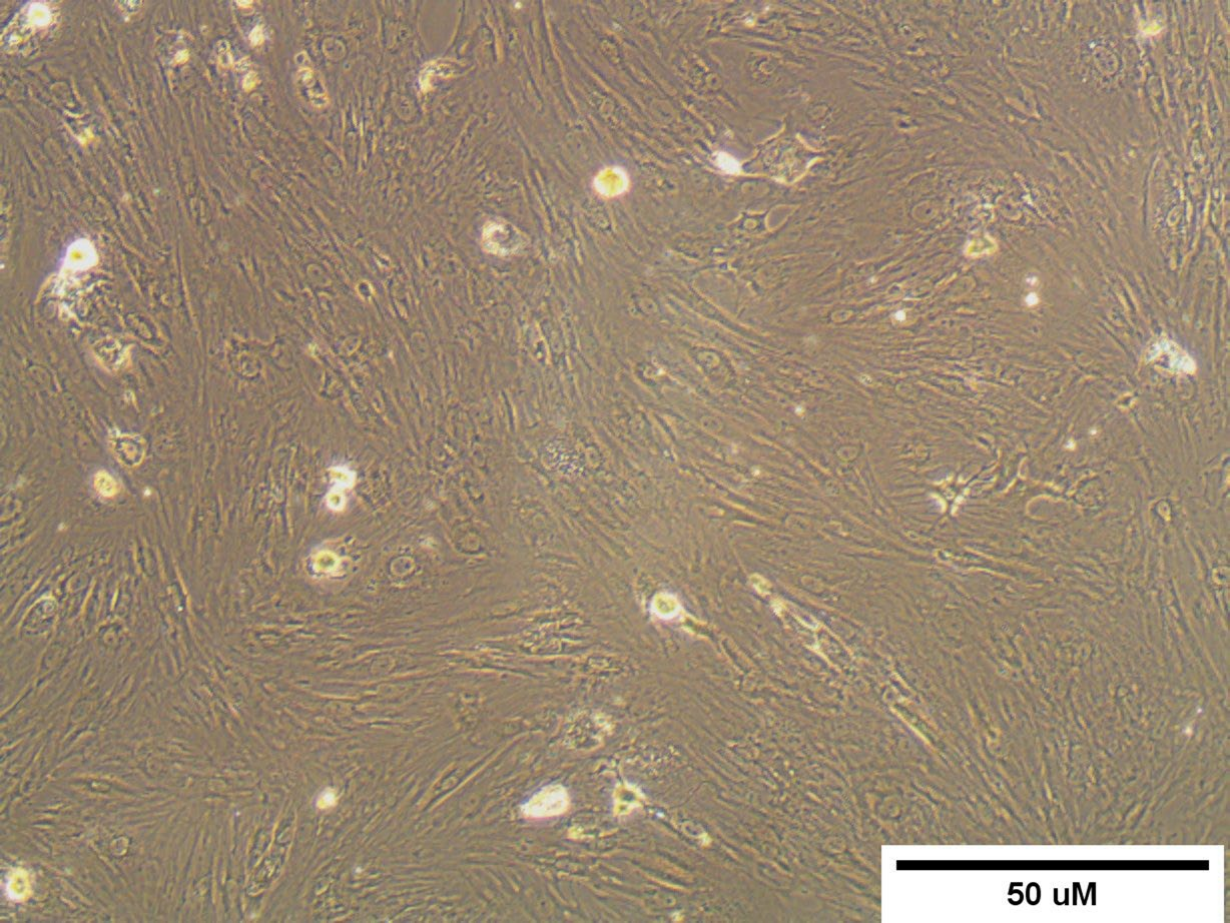
A microscopic observation of rat BM-MSCs of passage 3 displaying a confluent spindle-shaped cell sheet.

Bone marrow-derived mesenchymal stem cell cultures appeared as an adherent spindle-shaped cell sheet upon inverted microscope examination, as indicated in Fig. 1.

**Fig. 2:**
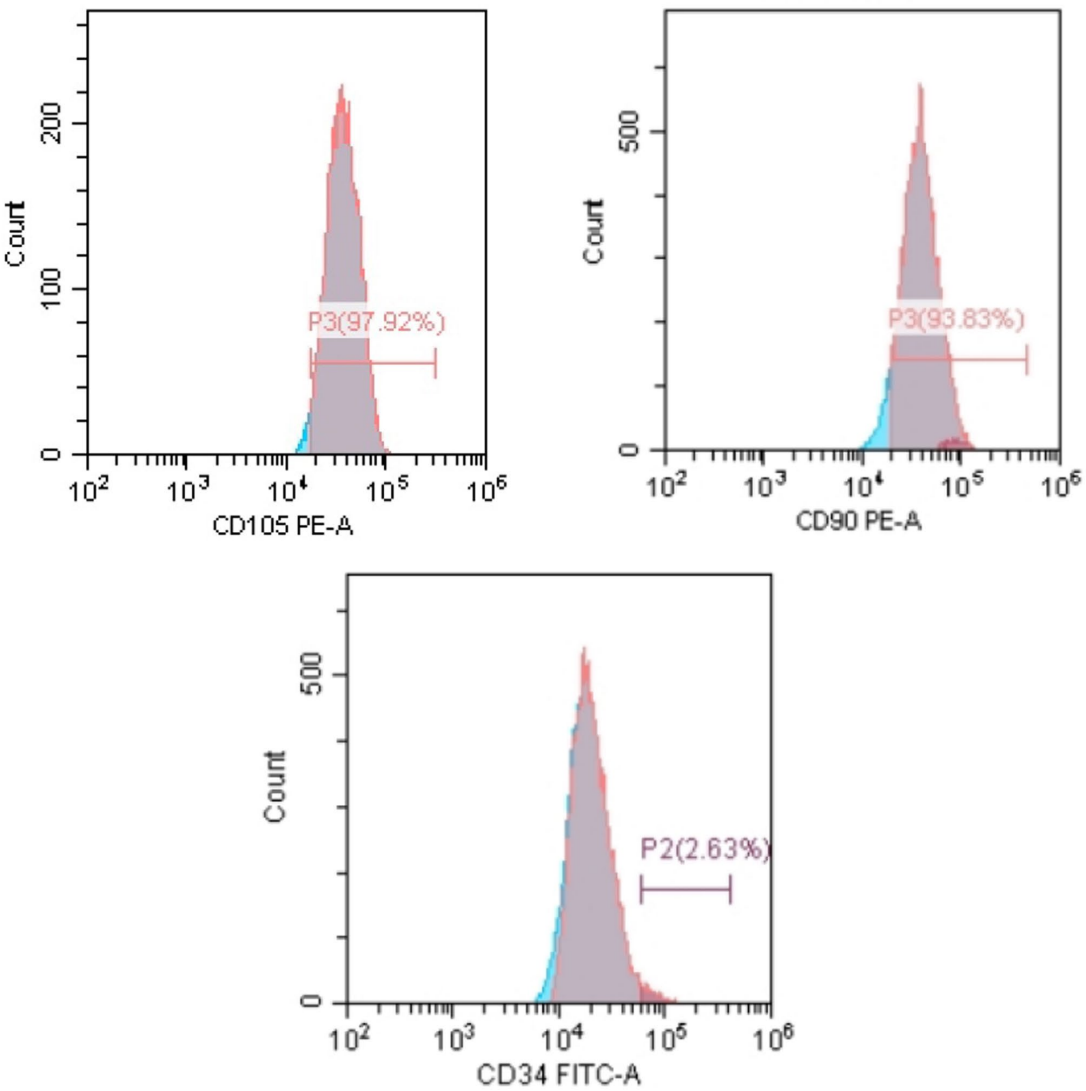
Flow cytometry identification of BM-MSCs demonstrating the expression of CD105 (97.92%), CD90 (93.8%), and CD34 (2.6%).

Flow cytometry analyses of BM-MSCs demonstrated positive immunostaining of MSC-specific surface antigen CD105 (97.92%) and CD90 (93.83%), and null immunostaining of hematopoietic stem cell antigen CD34 (2.63%) as depicted in Fig. 2.

### The regenerative impact of BM-MSCs on rat corneal epithelia

**Fig. 3:**
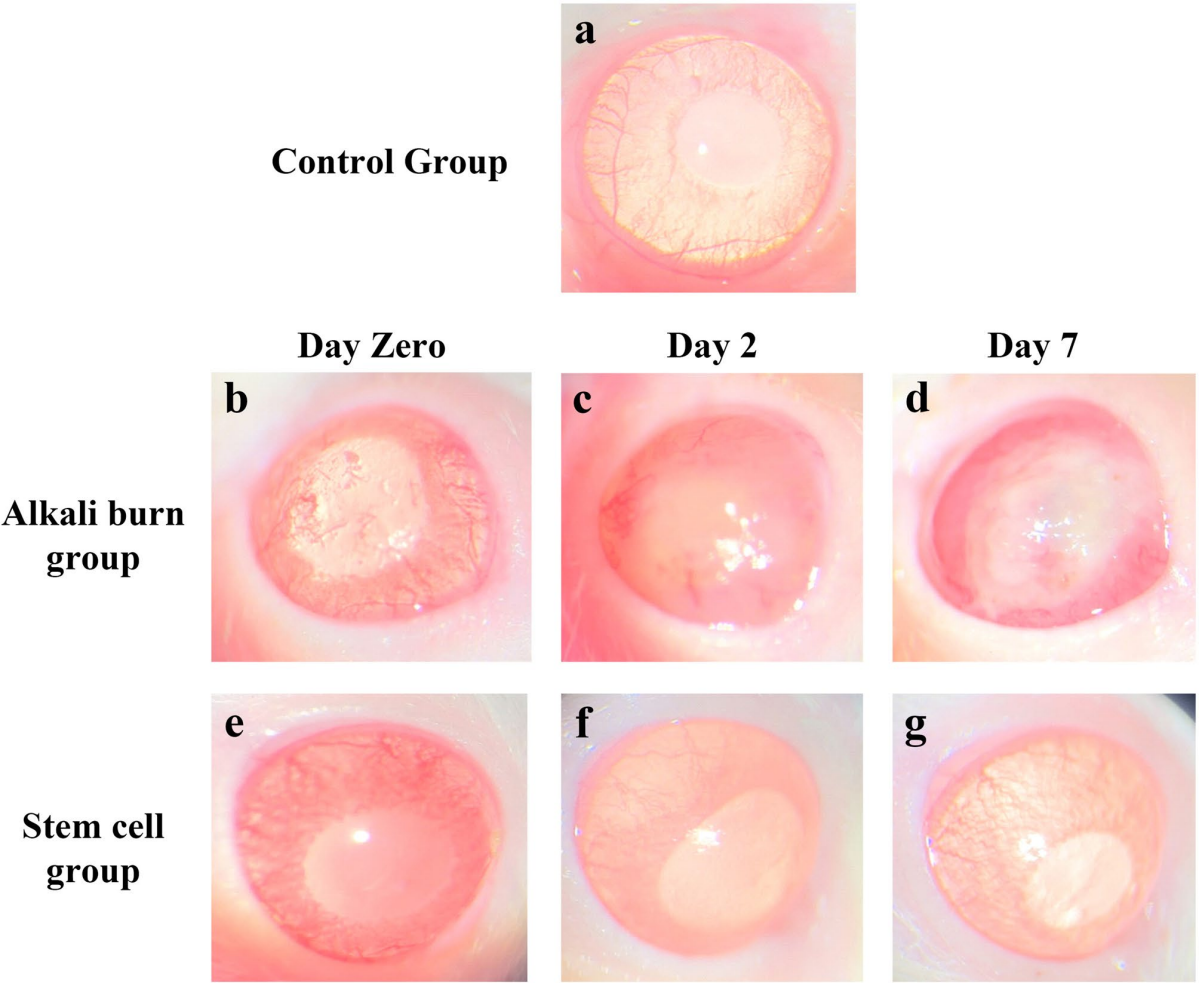
Representative slit-lamp images of rat cornea of (**a**): The control group, (**b**,** c** & **d**): The alkali burn group on day zero, 2, and 7 of induction, respectively, and (**e**,** f** & **g**): Stem cells group on day zero, 2, and 7 of induction, respectively.

Figure 3 displays the rat corneal surface examination by slit lamp after alkali burn induction and BM-MSCs treatment, showing the degree of opacity and neovascularization. Statistical analysis of neovascularization and opacity grading of different groups is displayed using scatter-bar charts (Fig. 5a and b, respectively). On the day of induction, loss of corneal luster, central corneal haziness, and irregular corneal surface were observed in both alkali burn and stem cells groups (Fig. 3b and e, respectively), when contrasted with the normal corneal surface of the control group (Fig. 3a). Moreover, the neovascularization grading was 3 ± 0.7 in the alkali burn group and (2.8 ± 0.44) in the stem cells group, whereas the opacity grading was (2.2 ± 0.44) and (1.6 ± 0.89), respectively. Two days after induction, corneal alkali-burned rats showed severe opacity (2.6 ± 0.89) and stromal hemorrhage (Fig. 3c) associated with severe neovascularization (3.6 ± 0.54). On the other hand, neovascularization was nearly absent (1.6 ± 0.89) and faint corneal opacity (1 ± 0.7) was observed in most rats of stem cells group (day 2), however irregular corneal surfaces were still observed (Fig. 3f). At the end of the experiment (7 days post-induction), the rat’s cornea in the alkali burn group revealed loss of corneal luster and early signs of corneal melting, as shown in Fig. 3d. This was accompanied by extremely severe neovascularization (3.8 ± 0.44) and a remarkable opaque corneal surface with extremely dense opacity (3.6 ± 0.54). Interestingly, the stem cells group revealed normal corneal structure with complete absence of both neovascularization and opacity in most rats of the group on day 7 after induction, as shown in Fig. 3g. This was reflected by a significant reduction (*p* < 0.05) in neovascularization grading (0.8 ± 0.44) and opacity grading (0.3 ± 0.68), compared to the alkali burn group (on day zero and 7), and the stem cells group on day zero.

### Fluorescein staining

**Fig. 4:**
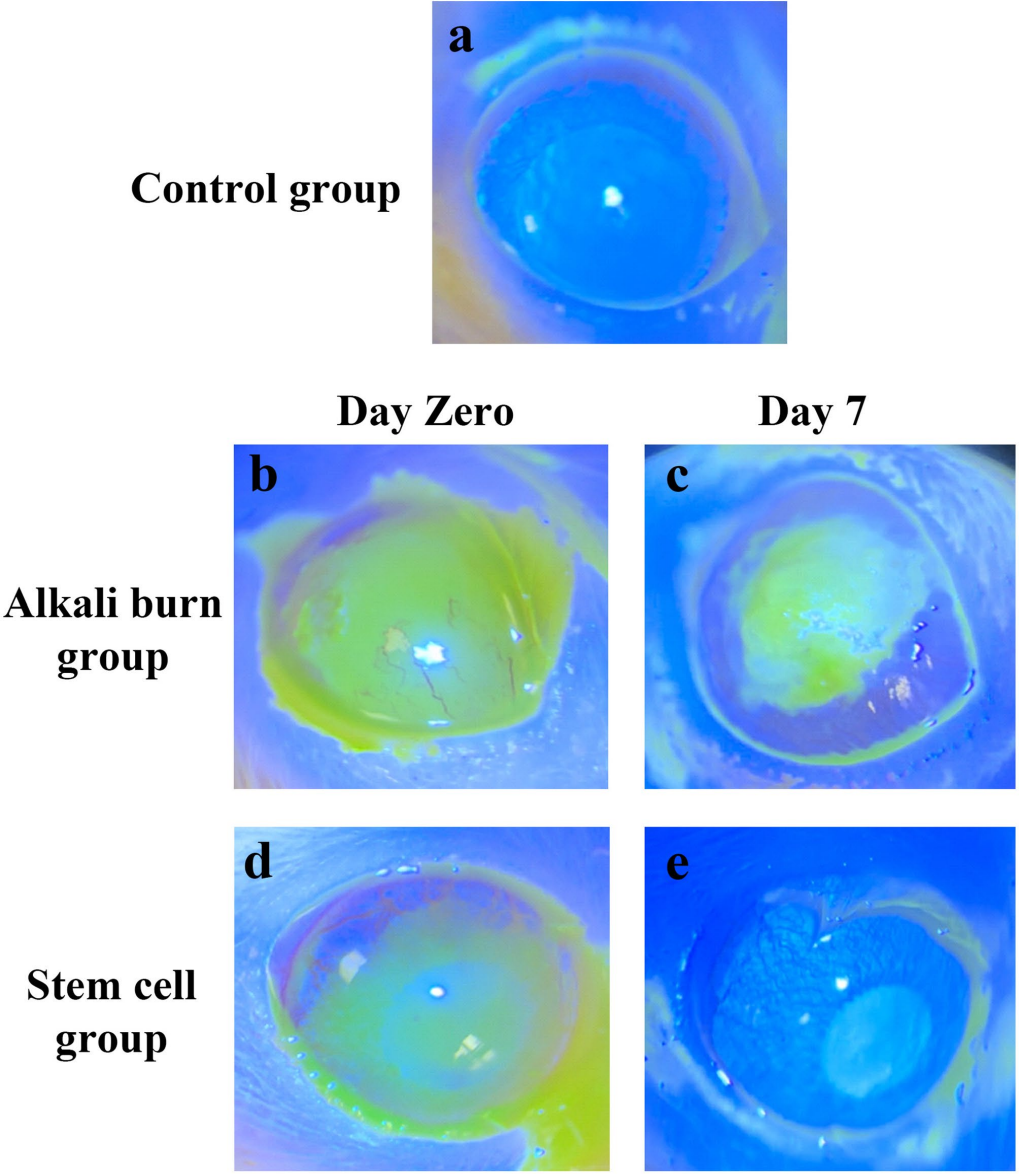
Representative photographs showing fluorescein staining of cornea from (a): the control group, (b & c): the alkali burn group on day zero and day 7 of induction, respectively, (d & e) the stem cells group on day zero and day 7 of induction, respectively.

**Fig. 5:**
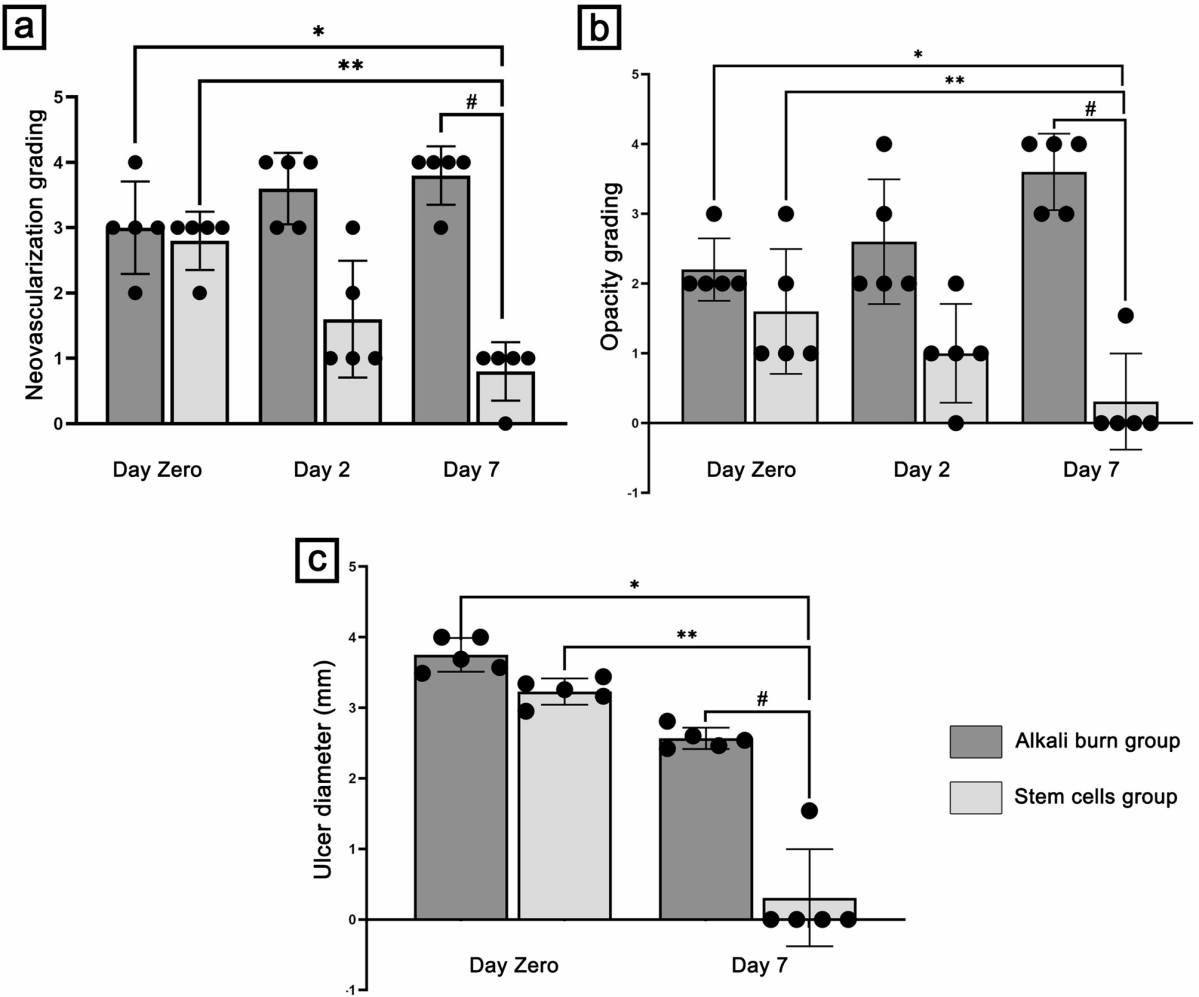
Scatter-bar charts displaying (**a)**: neovascularization grading, (**b)**: opacity grading, and (**c)**: ulcer diameter (mm), in different groups. Data are presented as mean ± S.D. (**n = 5 per group**). *: represents a significant change at *p* < 0.05 as compared to the alkali burn group on day zero, **: represents a significant change at *p* < 0.05 as compared to the stem cells group on day zero, **#**: represents a significant change at *p* < 0.05 as compared to the alkali burn group on day 7.

Figure 4 illustrates the photographs of fluorescein staining of the corneal surface of the different studied groups. On the day of induction, corneal surfaces of alkali burn rats revealed a positive fluorescein staining, with corneal ulcer diameter of 3.74 ± 0.24 mm and almost complete corneal surface loss (Figs. 4b and 5c), compared to the control group displaying a negative fluorescein staining (Fig. 4a). Similarly, the stem cells group revealed a positive fluorescein staining of corneal surface on the day of induction with corneal ulcer diameter of 3.22 ± 0.18 mm (Figs. 4d and 5c). Surprisingly, the cornea of the stem cell group displayed less damage compared with the alkali burn rats on the day of induction, indicating the early therapeutic response of stem cells within a few hours of administration. After 7 days of induction, the corneal surface of alkali burn rats showed positive fluorescein staining with a central corneal ulcer diameter of 2.56 ± 0.15 mm, indicating irregular corneal healing (Figs. 4c and 5c). On the contrary, after 7 days of induction, most corneal surfaces in stem cells group exhibited negative fluorescein staining, accompanied by a significant decrease (*p* < 0.05) in corneal ulcer diameter (0.3 ± 0.68 mm), as compared to alkali burn group (on day zero and 7), and stem cells group on day zero (Figs. 4e and 5c). This indicates regular corneal surface healing and the absence of any corneal defect.

### Biochemical findings

Data illustrated in Table 2 shows a significant enhancement (*p* < 0.05) in the inflammatory marker (TNF-α), inflammatory chemokine (IL-8), angiogenetic markers (VEGF and MMP-2) levels, and antioxidant enzyme (SOD) activity in the cornea homogenate samples of the alkali burn group relative to the control group. On the opposite side, subconjunctival infusion with BM-MSCs brought about a significant decline (*p* < 0.05) in TNF-α, IL-8, VEGF, and MMP-2 levels, along with almost recovery of the activity of SOD to normal value in the corneal tissue homogenate samples, as compared to the alkali burn group.

**Table 2:** Impact of stem cell treatment on TNF-α, IL-8, VEGF, MMP-2, and SOD protein levels in corneal tissue of alkali burn-induced rats.

	TNF-α (ng/g protein)	IL-8 (ng/g protein)	VEGF (ng/g protein)	MMP-2 (ng/mg protein)	SOD (ng/g protein)
Control group	31.41 ± 13.09	12.12 ± 1.14	87.93 ± 14.5	9.43 ± 0.89	9.02 ± 0.81
Alkali burn group	111.65 ± 18.21*	30.85 ± 1.59*	145.4 ± 20.51*	12.72 ± 0.2*	22.88 ± 3.42*
Stem cells group	44.22 ± 12.22**	18.32 ± 1.72**	116.4 ± 8.92**	9.51 ± 1.11**	14.1 ± 3.46**

Data are indicated as mean ± S.D. (*n* = 6 per group), *: represents a significant change at *p <* 0.05 by contrast with the control group, **: represents a significant change at *p <* 0.05 by contrast with the alkali burn group.

### Molecular outcomes

#### Gene expression patterns

**Fig. 6:**
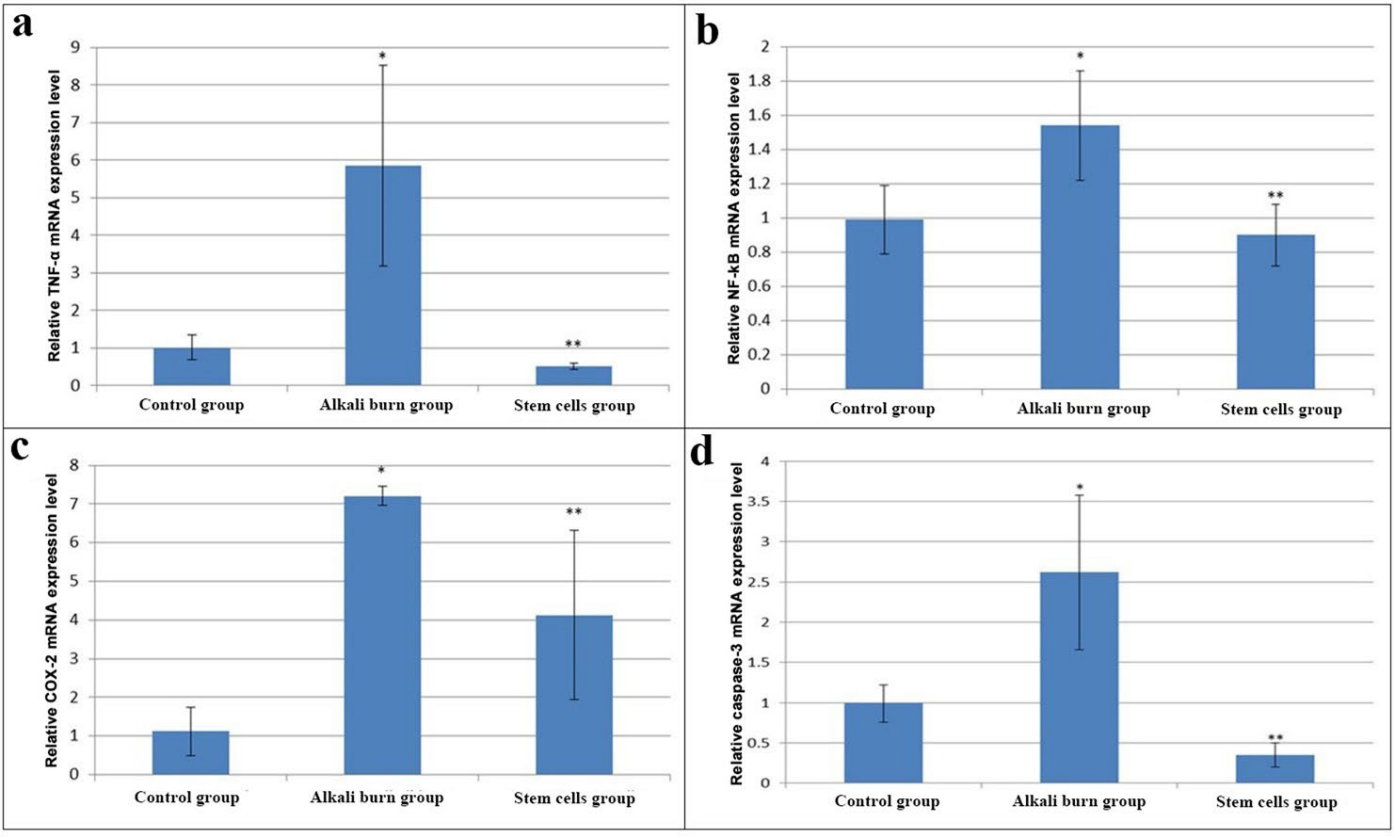
Relative mRNA expression of **a**: TNF-α, **b**: NF-kB, **c**: COX-2, and **d**: caspase-3. Data are presented as mean ± S.D. (*n* = 3 per group). *: represents a significant change at *p <* 0.05 *versus* the control group, **: represents a significant change at *p <* 0.05 *versus* the alkali burn group.

Figure 6 represents the influence of BM-MSCs treatment on the mRNA transcriptional level of TNF-α, NF-kB, COX-2, and caspase-3 in the LSCD rat model. Alkali burn-induced rats experienced a significant elevation (*p* < 0.05) of corneal TNF-α, NF-kB, COX-2, and caspase-3 mRNA expression levels *versus* the control group. On the other side, stem cell-treated rats displayed a significant down-regulation (*p* < 0.05) of corneal TNF-α, NF-kB, COX-2, and Caspase-3 gene expression levels as compared to the untreated alkali burn group.

#### Western blot results

**Fig. 7:**
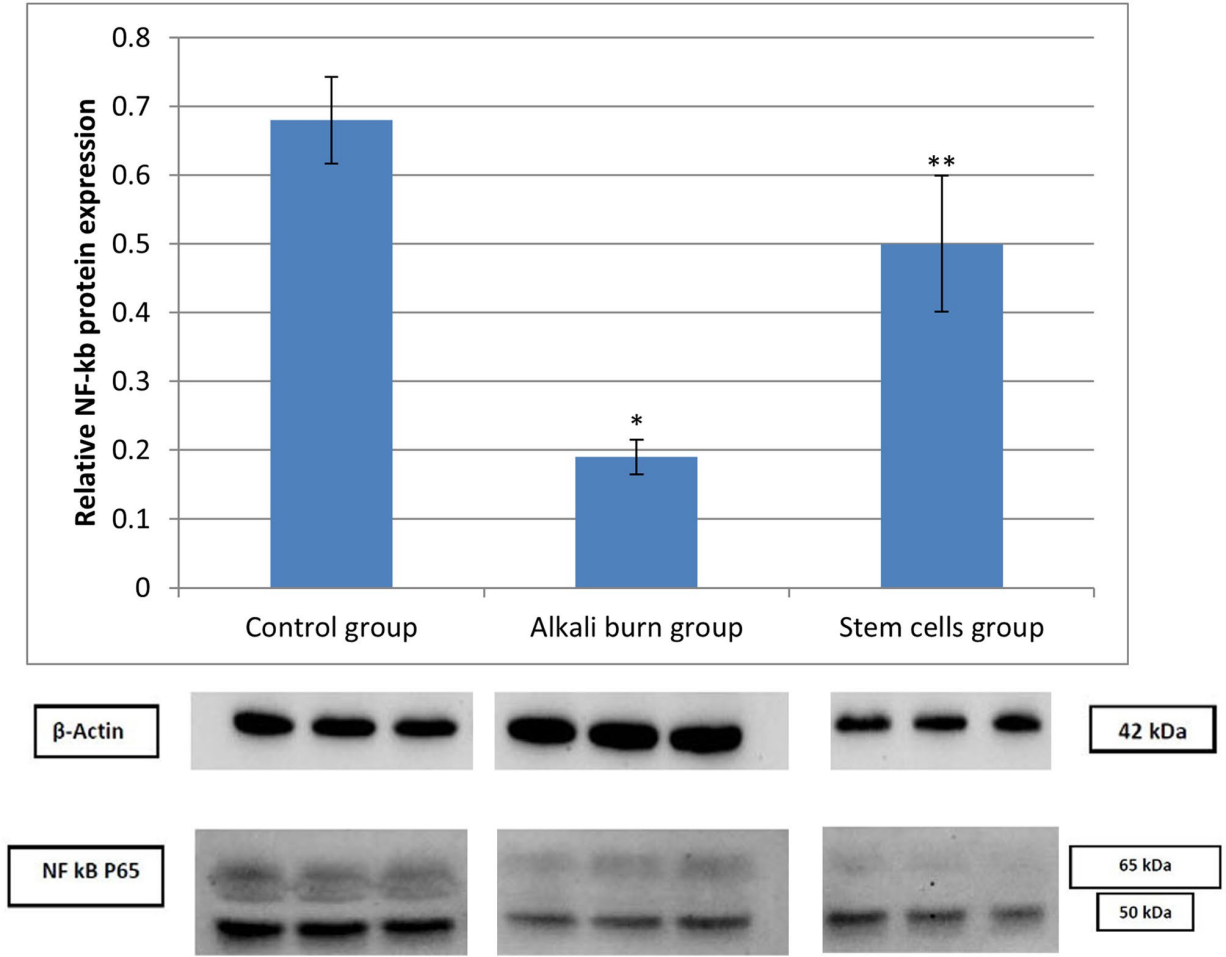
Protein expression patterns of NF-kB in the different studied groups. Data are displayed as mean ± S.D. (*n* = 3 per group). *: implies a significant change at *p* < 0.05 as opposed to the control group, **: implies a significant change at *p* < 0.05 as opposed to the alkali burn group. Original uncropped blots are presented in Supplementary Figures S1 & S2.

Figure 7 demonstrates the level of NF-kB protein expression assessed by western blot relative to β-actin (housekeeping protein) in the different experimental groups. The alkali burn group revealed a significant down-expression of NF-kB protein, contrary to the control group. Whereas stem cell treatment restored the NF-kB protein expression level to almost the normal level, contrary to the alkali burn group.

#### Histological findings

**Fig. 8:**
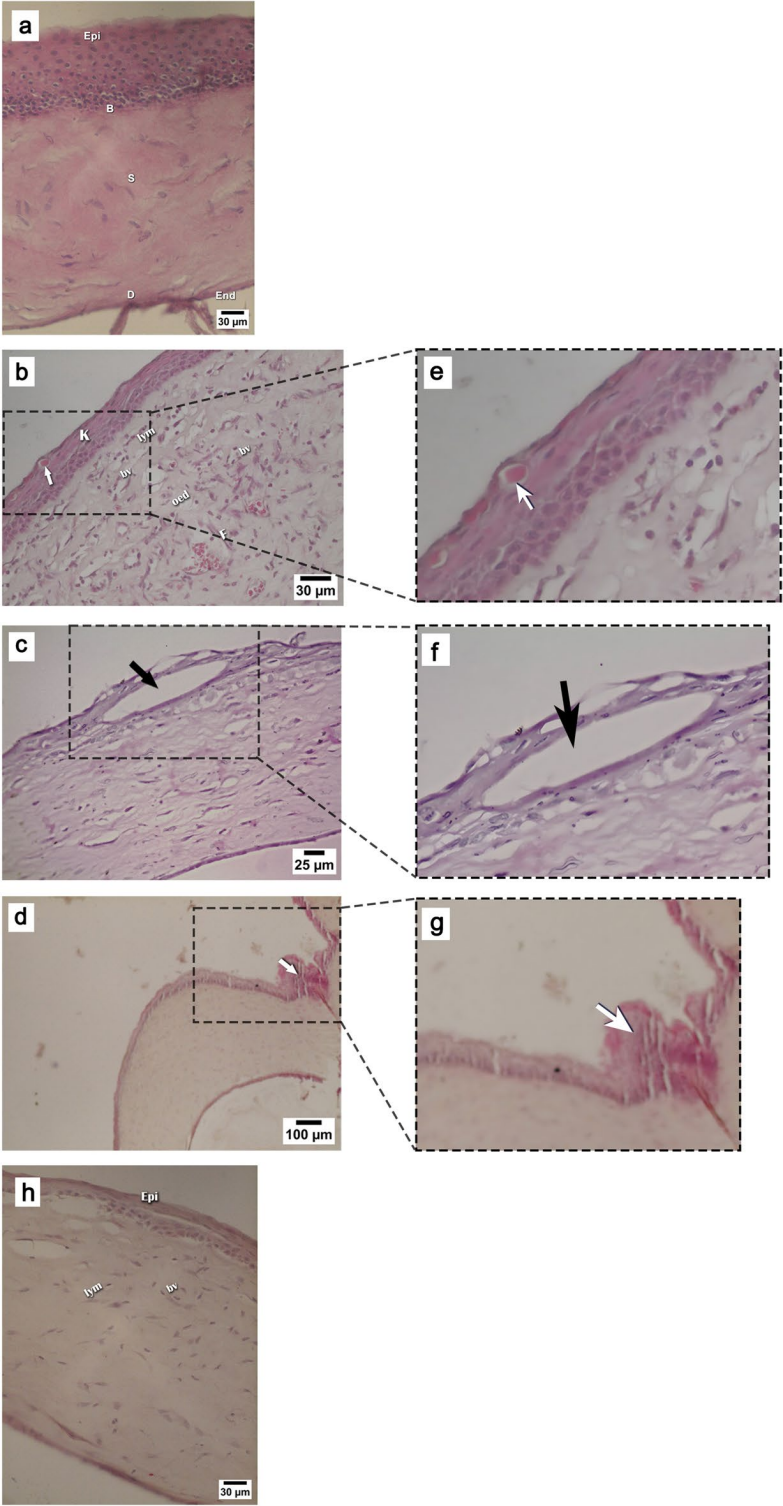
Microscopic images of hematoxylin and eosin-stained corneal tissue sections attained from (**a**): Control group revealing normal corneal layers; Epithelial layer (Epi), Basal membrane (B), Stroma (S), Descemet’s Membrane (D), and Endothelium (End); (**b**) The alkali burn group revealing hyperkeratosis (K) in the epithelium with dyskeratotic cells (arrows), stroma revealed dense lymphocytic infiltrate (Lym), numerous blood vessels (bV) with interstitial oedema (oed) and fibroblasts (F); (**c**) The alkali burn group displaying severe vacuolation of corneal epithelium (arrow) (**d**) The alkali burn group displaying conjunctival epithelial cells invading the cornea surface (arrow); (**h**) The stem cells group demonstrating an improvement in surface epithelium status (Epi), mild lymphocytic infiltrate (lym) and reduced of blood vessels (bv); (**e**) Digital magnification of the local area indicated by the arrow in (**b**), emphasizing dyskeratotic cells. (**f**) Digital magnification of the local area indicated by the arrow in (**c**), highlighting severe vacuolation of the corneal epithelium. (**g**) Digital magnification of the local area indicated by the arrow in (**d**), illustrating conjunctival epithelial cell invasion over the corneal surface.

Figure 8 demonstrates the histological characteristics of H&E-stained corneal tissue sections attained from the different experimental groups. Histological examination of the corneal tissue of the control group displayed normal epithelial and stromal integrity (Fig. 8a). On the opposite hand, the microscopic investigation of the corneal tissue of an alkali burn-induced rat revealed loss of normal epithelial and stromal integrity. Moreover, the epithelium showed hyperkeratosis and scattered dyskeratotic cells (Fig. 8b), as well as a severe vacuolation of corneal epithelium (Fig. 8c), The underlying stroma exhibited dense inflammatory infiltrate mainly lymphocytic with some neutrophils, evident stromal oedema, stromal scarring and fibroblasts (Fig. 8b), besides marked neovascularization is observed with an apparent invasion of the corneal surface with conjunctival epithelial cells (Fig. 8d), confirming the development of LSCD. The photomicrograph of corneal tissue of the stem cells group displayed a remarkable improvement in the ocular surface epithelial integrity, reduced density of the inflammatory cell infiltrate with decreased interstitial stromal edema, and diminished neovascularization (Fig. 8h).

#### Immunohistochemical outcomes

**Fig. 9:**
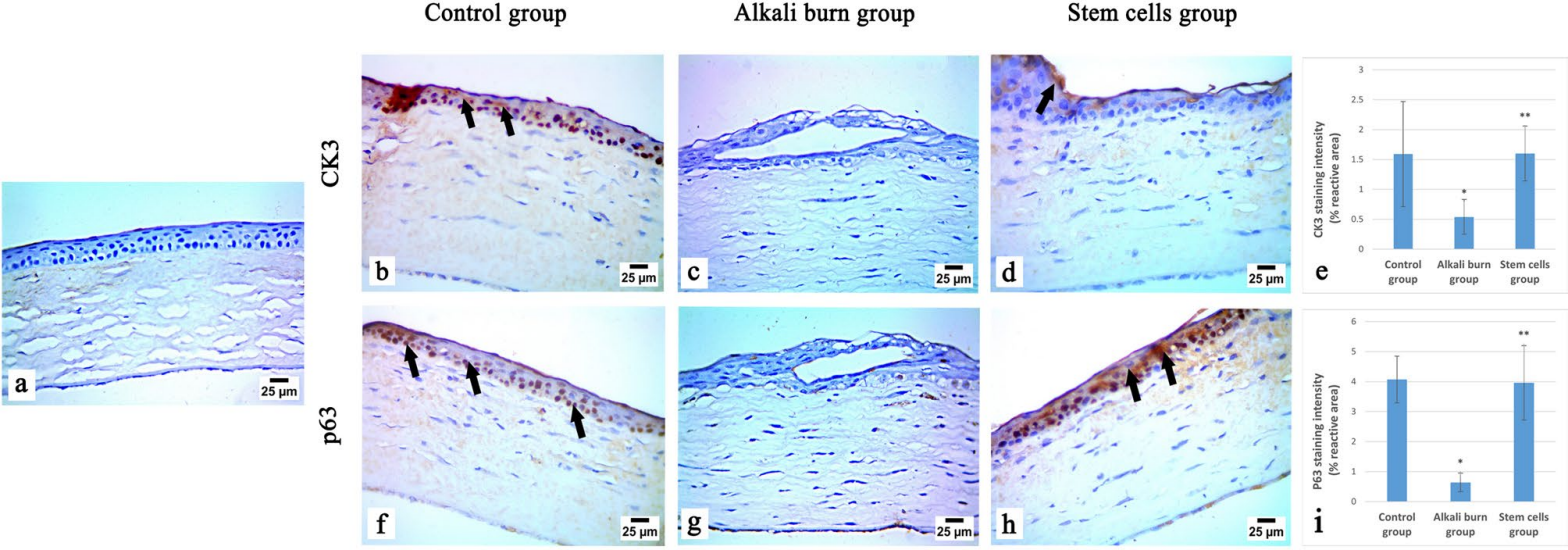
Immunohistochemical analysis of CK3 and p63 in the central cornea epithelial tissue sections of different groups. (**a**) negative control (without primary antibody), **(b)** the control group showing moderate CK3 expression (arrows), (**c**) the alkali burn group demonstrating negative CK3 expression, (**d**) the stem cells group showing moderate CK3 expression (arrow), (**f**) the control group showing strong p63 expression (arrows), (**g**) the alkali burn group showing negative p63 expression, (**h**) the stem cells group revealing strong p63 expression (arrows), (x400). (**e & i**) represents the percentage of CK3 and p63 immunoreactivity, respectively, in all experimental groups. Data are displayed as mean ± S.D. (*n* = 5 per group). *: implies a significant change at *p* < 0.05 *versus* the control group, **: implies a significant change at *p* < 0.05 *versus* the alkali burn group.

CK3 and p63 protein expressions in the central corneal epithelial tissue were determined by the immunohistochemical technique to evaluate the degree of corneal epithelial and limbal epithelial stem cells differentiation. Figure9 depicts the photomicrographs of p63 and CK3 immunostaining of the corneal tissue of the different groups. The corneal epithelial tissue section of the control group revealed a strong expression of p63 and a moderate expression of CK3 proteins, indicating normal corneal epithelial differentiation. Whereas the alkali burn group showed a significant decrease (*p* < 0.05) in the number of p63 and CK3 immunoreactive cells, as compared to the control group, indicating corneal epithelial damage and limbal stem cell loss. Remarkably, the stem cells group exhibited a significant elevation (*p* < 0.05) in p63 and CK3 expression *versus* the alkali burn group, as indicated by strong p63 and moderate CK3 immunoreactivity in the basal epithelial layers, indicating the restoration of normal corneal epithelial differentiation and limbal stem cell regeneration.

## Discussion

The current investigation was constructed to appraise the therapeutic efficiency of BM-MSCs in alleviating LSCD elicited by acute corneal alkali injury in rats. This goal was accomplished by determining the biochemical, molecular, and histological changes provoked by BM-MSCs treatment *via* investigating the biomarkers implicated in inflammation, neovascularization, and apoptosis. Specifically, BM-MSCs accelerate corneal wound healing through rapid re-epithelialization, attenuating oxidative stress and inflammation, and enhancing corneal clarity more effectively than adipose-derived MSCs^25^. In addition, they are more applicable for both autologous and allogeneic treatments owing to their ease of isolation and culture, making them perfect candidates for corneal wound-healing approaches^26^. Hence, they have been chosen in the current study.

Microscopic examination and flow cytometry analysis of the BM-MSCs validated the main characteristics of MSCs. These outcomes are in line with the findings of the research of Mahmoud et al.^16^. Ocular surface examination using a slit lamp and fluorescein staining of the corneal surface of alkali burn-induced rats demonstrated corneal opacity and neovascularization, contrary to the control group. These data fit those of Luisi et al.^27^. On the other side, stem cell treatment resulted in the alleviation of corneal scars and the absence of both corneal opacity and neovascularization, contrary to the corneal surface of the alkali burn-induced rats. Moreover, the corneal epithelium restored its integrity, indicating the regenerative ability of BM-MSCs in stimulating wound healing and reconstructing the corneal epithelial surface. These observations corroborate earlier studies of Ye et al.^13^ and Yao et al.^15^. Surprisingly, the cornea of the stem cell group displayed less damage compared with the alkali burn rats on day zero. This observation may be attributed to the early protective or therapeutic effect of stem cells administered immediately after the injury, especially since the imaging of all groups (alkali-burned group and stem cell group) was captured 6 h post-injury, and post-MSCs treatment on day zero. It has been reported that mesenchymal stem cells may exert early anti-inflammatory or cytoprotective effects within a few hours of administration *via* paracrine signaling^28^, which could explain the reduced corneal damage seen in the stem cell group on day zero.

The present results uncovered a significant up-regulation of both mRNA expression and protein levels of TNF-α in the corneal tissue of the alkali burn group, in contrast with the control group. These findings match those of Cade et al.^29^. Similarly, our study observed a significant enhancement in IL-8 protein level in the corneal tissue homogenate of the alkali burn group on comparison with the control group. This outcome aligns with that of Santacruz et al.^30^, who recorded a significant elevation in IL-8 levels in tear samples of human corneal keratitis, compared to the non-infected ones. Alkali burn causes severe corneal epithelium damage, triggering an immediate liberation of inflammatory cytokines and chemokines from the basal membrane of the corneal epithelium to the site of injury. This initiates stromal inflammation and recruits immune cells like T-cells, thereby intensifying the inflammatory reaction^31^. Notably, it has been reported that TNF-α induces the expression of IL-8 by activating the NF-kB pathway^32^. This mechanism explains the elevated levels of TNF-α and IL-8 observed in corneal tissue following alkali injury. Concerning the stem cells group, both mRNA expression and protein levels of TNF-α in corneal tissue exhibited a significant down-expression as compared to the alkali burn group, consistent with the findings of Yao et al.^15^. Likewise, stem cell infusion in corneal alkali burn rats elicited a significant drop in IL-8 level in corneal tissue homogenate, in line with the results of Tao et al.^33^. These findings prove the anti-inflammatory effect of BM-MSCs in LSCD-stimulated cornea, suggesting that they suppress the liberation of pro-inflammatory cytokines from the basal membrane of the corneal epithelium and stimulate the regeneration of the damaged corneal cells caused by alkali burn. It has been found that the augmented levels of inflammatory cytokines following tissue injury could activate MSCs and stimulate their anti-inflammatory phenotypic transition. This leads to the secretion of anti-inflammatory factors such as TNF-α-stimulated gene/protein 6 (TSG-6) and inhibition of T cell proliferation, leading to alleviation of the inflammatory response^34^.

The present investigation reported a significant hike in the corneal VEGF and MMP-2 levels in the alkali burn group *versus* the control group, consistent with the findings of Hakami et al.^35^ and Oh et al.^36^, respectively. These results could be ascribed to the acute inflammation elicited by alkali burn, which subsequently promotes angiogenesis. It has been proven that TNF-α activates the NF-kB signaling cascade^37^, which subsequently stimulates the transcriptional activation of VEGF^38^. The corneal neovascularization is initiated by triggering leukocyte attraction, followed by degeneration of the endothelial basement membrane and extracellular matrix (ECM), leading to the invasion of endothelial cells and formation of vascular sprouts^39,40^. Additionally, MMP-2 has been reported to motivate the production of angiogenic factors, including VEGF^41^. Therefore, it can be concluded that inflammation is the main regulator of the neovascularization process^42^.

The present data elucidated that BM-MSCs infusion in the alkali-injured cornea of rats produced a significant diminution in corneal VEGF and MMP-2 levels as compared to the alkali burn group. These outcomes are supported by the findings of Yao and colleagues^15^ and Ma et al.^43^, respectively. This study declared that the possible downregulation of VEGF and MMP-2 mRNA expression levels following MSCs treatment could be attributed to the upregulation of mRNA levels of thrombospondin-1 (TSP-1), which is recognized to inhibit angiogenesis by stimulating the death of endothelial cells and downregulating the expression of VEGF and MMPs. The detailed mechanism underlying the anti-angiogenic action of MSCs could be explained as the inflammatory signaling from injured corneas triggers the activation of pro-inflammatory factors, which in turn stimulate MSCs to reduce inflammation *via* releasing anti-inflammatory factors such as TSG-6^44^. TSG-6 is known to restrain inflammation by reducing neutrophil infiltration and protease production into the cornea, thus decreasing corneal opacity and neovascularization^45^. Hence, MSCs are involved in the anti-angiogenesis of the injured cornea, which is accredited to their anti-inflammatory effect.

Corneal alkali injury stimulates oxidative stress by eliciting the production of ROS in the cornea, followed by excessive corneal inflammation, neovascularization, and scar formation^46^. The current approach demonstrated that the corneal SOD activity was significantly increased following alkali injury of the rat cornea. Such increase in SOD activity may be ascribed to the acute inflammation and excessive oxidative stress stimulated by alkali burn leading to a sudden and immediate elevation in SOD activity to scavenge the excessive production of free radicals in an attempt to protect the corneal epithelia from the detrimental effect of oxidative stress in the early stage after corneal injury, especially the corneal samples were collected after 7 days only from the injury, which may be later followed by a decline in SOD activity as the oxidative stress levels continue to increase. This finding is in accordance with the results of the study of Sahreen and colleagues^47^ who reported a significant enhancement in SOD activity in the infected (swim bladder, intestine, and liver) of the Indian catfish with *Isoparorchis hypselobagri* parasite, as compared to the non-infected one. These investigators explained the elevated SOD activity by the excessive increase in oxidative stress levels. Since SOD represents the first line of defense to confine the destruction caused by ROS. So, SOD elevation could be an indicator for increased oxidative stress levels. Also, these findings fit those of Assady et al.^48^ who recorded increased SOD activity in sheep liver afflicted with Fasciola spp. parasites.

Stem cell infusion in the alkali-injured cornea significantly recovered the SOD activity to almost a normal value, as compared to the alkali burn group. This indicates the diminished level of oxidative stress after MSCs infusion, leading to a decrease in the degree of inflammation and restoring the normal antioxidant defense system, including SOD. This result is consistent with that of Jung and colleagues^49^ reported that MSCs treatment in a chronic large intestine inflammation mouse model successfully recovered the SOD activity to nearly its normal value, along with decreasing the levels of ROS as compared to the diseased group. The mechanism behind the ability of MSCs to exert their antioxidant effect is related to stimulating the expression of Heme Oxygenase 1 (HO-1) and SOD, which shield the cells from oxidative stress destruction *via* activating the nuclear factor erythroid-2-related factor 2 (Nrf2)/antioxidant response element (ARE) pathway. Upon activation of the Nrf2/ARE pathway by MSCs treatment, Kelch-like ECH-associated protein 1 (Keap1) is degraded by ubiquitination, resulting in activation of Nrf2 and its dissociation from Keap1 in the cytoplasm, followed by its nuclear transfer, where it combines with ARE to stimulate the transcriptional activity of downstream genes such as HO-1, catalase, and SOD^50^.

The present data revealed a significant upregulation of NF-kB mRNA expression in the alkali burn group, contrary to the control group, which is in agreement with the study of Tobita et al.^51^. This could be attributed to the severe inflammation caused by alkali burn of rat cornea, triggering the production of TNF-α, and subsequently activating the NF-kB signaling pathway. Once TNF-α binds to its receptor, TNF receptor-associated factor (TNFR1), trimerization occurs, resulting in the recruitment of adapter molecules, which subsequently recruit and activate inhibitory kappa B Kinase (IKK). The activated IKK phosphorylates the inhibitory kappa B beta (IκBα), leading to its degradation. This leads to the release of NF-κB from IκB, followed by nuclear transfer of NF-κB, where it induces the transcriptional activation of target genes^52^.

Our study recorded a significant downregulation of NF-kB protein expression in the alkali burn group *versus* the control group, in contrast to the upregulation observed at its mRNA level. This result is supported by Prabahar et al.^53^, who reported the divergence between the mRNA and protein levels, resulting from the complexities of gene expression and translational control at the protein level, and variable experimental conditions. They recorded weak or no correlation between mRNA and protein levels in 15 genes in the experimental canine Volumetric Muscle Loss (VML) wound healing model under different experimental conditions, including the time of taking samples and the wound zones.

Alkali-induced corneal injury is considered an acute inflammation model associated with a rapid-onset inflammatory response, including neutrophil infiltration, edema, vasodilation, and ROS production^54^. Although NF-κB mRNA expression was increased, the observed reduction in protein levels may be attributed to post-transcriptional regulation or enhanced proteasomal degradation of NF-κB subunits, mechanisms which may be activated under acute inflammatory conditions. Moreover, negative feedback mechanisms that trigger NF-κB downregulation at the protein level can occur during acute inflammation to regulate the response and promote its termination. These feedback loops can engage the production of microRNAs (like miR-146 and miR-155) in response to NF-κB activation, which then inhibit NF-κB protein expression by directly regulating the stability and translation of mRNA transcripts encoding key components of the NF-κB signaling cascade^55–57^. Termination of NF-κB transcriptional activity is mainly accomplished by the fact that NF-κB induces its own IκB family inhibitors. For instance, IκBα enters the nucleus, where it displaces NF-κB from the DNA and relocates it to the cytoplasm. Moreover, NF-κB can induce other negative regulators, including A20 and CYLD. In case of acute inflammation, these negative feedback loops usually lead to the inhibition of NF-κB to its normal level. On the contrary, in chronic inflammatory conditions, the persistent NF-κB-activating stimuli can override the inhibitory mechanisms, resulting in constitutive activation of NF-κB^58^. These mechanisms could explain why mRNA levels of NF-κB were elevated in our study, while protein levels were reduced due to enhanced degradation or translational suppression.

The present study denoted that stem cell infusion in the chemically injured cornea of rats significantly decreased NF-kB mRNA expression and restored its protein expression to nearly its normal levels relative to the control group. These findings fit those reported by Sayed et al.^59^. As previously described, NF-kB activation is mediated by TNF-α^53^. It has been proven that MSCs can alleviate inflammation by inhibiting the expression of inflammatory factors such as TNF-α^15^. So, such recovery of NF-kB expression level may be ascribed to the anti-inflammatory action of MSCs, resulting in the suppression of the NF-kB pathway.

In the present work, a significant upregulation of COX-2 mRNA expression levels was recorded in the alkali burn group *versus* the control group, which is supported by the results of Kim et al.^54^. In case of inflammation, the upregulation of COX-2 mRNA expression is triggered by TNF-α *via* activation of TNFR1 receptor, leading to the generation of Mitochondrial ROS (MitoROS), which consequently activates Protein Kinase C alpha PKCα/p38 (Mitogen-activated protein kinase) MAPK and c-Jun N-Terminal Protein Kinase(JNK)1/2 cascades to induce Forkhead box protein O1 (FoxO1) binding with the COX-2 promoter, leading to stimulating the COX-2 gene expression^60^.

Concerning the stem cells group, mRNA expression levels of COX-2 were significantly downregulated relative to the alkali burn group, which is approved by Medina et al.^61^. This could be explained by the ability of MSCs to restrain the liberation of pro-inflammatory factors such as TNF-α^15^, which is known to induce the transcriptional activation of COX-2^60^. Therefore, this could lead to a decreased mRNA expression level of COX-2 as a result of MSCs treatment.

The current outcomes demonstrated a significant over-expression of caspase 3 mRNA level in the alkali burn group, contrary to the control group, which concurred with that of Kim and colleagues^54^. This could be explained by the acute inflammation elicited in the alkali burn group, resulting in the corneal epithelium damage and triggering apoptotic cascades, resulting in the induction of caspase-3. It has been reported that the transcription of caspase-3 gene is activated by TNF-α as a result of inflammation. Once TNF-α combines with its receptor, TNF receptor 1 (TNFR1), this results in the assembly of TNF Complex IIb and the activation of Fas-associated death domain protein (FADD) and pro-caspase-8 to initiate the apoptotic signaling pathway, leading to the activation of downstream caspase-3 and initiation of apoptosis^62,63^.

On the contrary, stem cell injection in the alkali-injured cornea of rats resulted in a significant down-expression of caspase 3 transcriptional levels in the rat cornea *versus* the untreated alkali burn group, and which is documented by Zhang et al.^64^. This result confirms the anti-apoptotic effect of MSCs, likely due to their ability to attenuate inflammation by inhibiting pro-inflammatory mediators such as TNF-α, leading to the observed downexpression of caspase-3 *via* the apoptotic cascade, thereby restraining apoptosis. MSCs are known to modulate apoptotic pathways through several mechanisms, mainly by secreting bioactive components, including non-coding RNAs and chemokines that inhibit caspase activation in target cells^65^.

The histological examination of the alkali burn group indicated loss of normal epithelial and stromal integrity, dense inflammatory infiltration, stromal scarring, and marked neovascularization. Moreover, the obvious invasion of conjunctival epithelial cells into the avascular corneal surface confirmed the limbal barrier destruction and induction of LSCD, validating our model. These findings are in coherence with the study of Jiang et al.^14^. On the other hand, histological findings of the stem cells group revealed the recovery of the corneal epithelial integrity, reduced neovascularization, and decreased inflammatory cell infiltration as compared to the untreated alkali burn group. In addition, it revealed its ability to reduce the density of the inflammatory cell infiltrate. These observations coincide with those reported by Oh et al.^36^ and Ma et al.^43^.

Immunohistochemical staining of p63 and CK3 further confirmed the LSCD induction in the rat corneal epithelium. CK3 is a marker of differentiated corneal epithelial cells^66^, whereas p63 is a marker for limbal stem cells^67^. The alkali burn group demonstrated a significant decline in both markers compared to controls, indicating severe corneal epithelial damage and limbal stem cell depletion. These findings confirm the limbal barrier destruction and successful LSCD induction. On the contrary, the stem cells group revealed a significant increase in p63 and CK3 immunoreactive cells in the basal epithelial layers, compared to the alkali burn group, indicating partial regeneration of the limbal stem cell niche and re-establishment of corneal epithelial differentiation. These outcomes are consistent with the study by Rohaina et al.^68^.

## Conclusion

Overall, it can be concluded that subconjunctival injection of BM-MSCs could offer a promising remedy for LSCD by repairing damaged corneal epithelium, accelerating wound healing, and restoring the regenerative ability of damaged limbal stem cells. This preferential effect could be attributed to their anti-inflammatory, anti-angiogenic, and anti-apoptotic effects to suppress corneal inflammation, neovascularization, and apoptosis, which could render this stem cell-based therapy a clinical reality.

### Limitations of the study

Despite the promising results of the current study, some limitations must be acknowledged. First, we did not perform a cell-tracking assay to confirm the integration or differentiation of transplanted BM-MSCs into corneal epithelial cells. Therefore, we cannot verify whether the observed regenerative effects of MSCs were attributed to direct cellular regeneration or paracrine action on the local microenvironment. Moreover, measuring direct ROS markers would be valuable for confirming the ability of MSCs in restraining oxidative stress associated with corneal alkali injury. Also, immunostaining for conjunctival epithelial markers would be valuable to confirm that conjunctivalization occurs following alkali burn. Further studies are required to provide more preclinical evidence about the different mechanisms of BM-MSCs against LSCD before it can be considered for clinical application.

### Future perspective

This study sheds light on the prominent healing efficacy of BM-MSCs in treating LSCD and addresses the root cause of this condition. In the future, stem cell therapy could be a safe, less invasive alternative to corneal surgical interventions that lack long-term functionality and hold a risk for many complications, including graft rejection and infection.

## Supplementary Information

Below is the link to the electronic supplementary material.

Supplementary Material 1

Supplementary Material 2

Supplementary Material 3

## Data Availability

The datasets used and/or analyzed during the current study are available from the corresponding author on reasonable request.

## Abbreviations

BM-MSCs: Bone marrow-derived mesenchymal stem cells
COX-2: Cyclooxygenase-2
H&E: Hematoxylin & eosin
IL-8: Interleukin 8
LSCD: Limbal stem cell deficiency
MMP-2: Matrix metalloproteinase 2
NF-kB: Nuclear factor kappa B
ROS: Reactive oxygen species
SOD: Superoxide dismutase
TNF-α: Tumor necrosis factor-alpha
VEGF: Vascular endothelial growth factor

## References

[1] Singh, V. K., Kumari, N., Kusumesh, R. & Sinha, B. P. Limbal stem cell deficiency: demography, aetiology, and clinical presentation in Eastern India. *J. Family Med. Prim. Care*. **13**, 48–53 (2024). 38482302 10.4103/jfmpc.jfmpc_475_23 PMC10931896

[2] Cheung, A. Y. et al. Limbal stem cell deficiency: demographics and clinical characteristics of a large retrospective series at a single tertiary referral center. *Cornea* **40**, 1525–1531 (2021). 34050070 10.1097/ICO.0000000000002770

[3] Hu, J. C. W. & Trief, D. A narrative review of limbal stem cell deficiency & severe ocular surface disease. *Ann. Eye Sci.* **8**, 13–13 (2023).

[4] Moshirfar, M. et al. Turner syndrome: ocular manifestations and considerations for corneal refractive surgery. *J. Clin. Med.*. **11** (11), 6853 (2022). 36431330 10.3390/jcm11226853 PMC9692343

[5] Bian, Y. & Jurkunas, U. Ocular chemical injuries and limbal stem cell deficiency (LSCD): an update on management. *Int. Ophthalmol. Clin.* **64**, 31–48 (2024). 38525980 10.1097/IIO.0000000000000487

[6] Le, Q., Chauhan, T. & Deng, S. X. Diagnostic criteria for limbal stem cell deficiency before surgical intervention—a systematic literature review and analysis. *Surv. Ophthalmol.* **65**, 32–40 (2020). 31276736 10.1016/j.survophthal.2019.06.008 PMC6911822

[7] Deng, S. X. et al. Global consensus on the management of limbal stem cell deficiency. *Cornea* **39**, 1291–1302 (2020). 32639314 10.1097/ICO.0000000000002358

[8] Iyer, G., Srinivasan, B., Agarwal, S., Agarwal, M. & Matai, H. Surgical management of limbal stem cell deficiency. *Asia Pac. J. Ophthalmol. (Phila)*. **9**, 512–523 (2020). 33323706 10.1097/APO.0000000000000326

[9] Elhusseiny, A. M. et al. Current and emerging therapies for limbal stem cell deficiency. *Stem Cells Transl. Med.* **11**, 259–268 (2022). 35303110 10.1093/stcltm/szab028 PMC8968724

[10] Wu, C. H., Weng, T. F., Li, J. P. & Wu, K. H. Biology and therapeutic properties of mesenchymal stem cells in leukemia. *Int. J. Mol. Sci.*. **25**, 2527 (2024). 38473775 10.3390/ijms25052527 PMC10932140

[11] Tan, L., Liu, X., Dou, H. & Hou, Y. Characteristics and regulation of mesenchymal stem cell plasticity by the microenvironment — specific factors involved in the regulation of MSC plasticity. *Genes Dis.* **9**, 296–309 (2022). 35224147 10.1016/j.gendis.2020.10.006 PMC8843883

[12] Liu, S. et al. Immunosuppressive property of MSCs mediated by cell surface receptors. *Front. Immunol.* **11**, 538714 (2020). 10.3389/fimmu.2020.01076 PMC7399134 32849489

[13] Ye, J., Yao, K. & Kim, J. C. Mesenchymal stem cell transplantation in a rabbit corneal alkali burn model: engraftment and involvement in wound healing. *Eye* **20**, 482–490 (2005). 10.1038/sj.eye.6701913 15895027

[14] Jiang, T. S. et al. Reconstruction of the corneal epithelium with induced marrow mesenchymal stem cells in rats. *Mol. Vis.* **16**, 1304 (2010). 20664793 PMC2905634

[15] Yao, L. et al. Role of mesenchymal stem cells on cornea wound healing induced by acute alkali burn. *PLoS One*. **7**, e30842 (2012). 22363499 10.1371/journal.pone.0030842 PMC3281878

[16] Mahmoud, N. S. et al. Role of nanoparticles in osteogenic differentiation of bone marrow mesenchymal stem cells. *Cytotechnology* **72**, 1–22 (2020). 31722051 10.1007/s10616-019-00353-y PMC7002803

[17] Woodbury, D., Schwarz, E. J., Prockop, D. J. & Black, I. B. Adult rat and human bone marrow stromal cells differentiate into neurons. *J. Neurosci. Res.* **61**, 364–370 (2000). 10931522 10.1002/1097-4547(20000815)61:4<364::AID-JNR2>3.0.CO;2-C

[18] He, J. et al. Diethyl blechnic exhibits Anti-Inflammatory and antioxidative activity via the TLR4/MyD88 signaling pathway in LPS-Stimulated RAW264.7 cells. *Molecules 2019*. **24**, 4502 (2019). 10.3390/molecules24244502 PMC6943418 31835323

[19] Hussien, H. M. et al. Neuroprotective effect of Berberine against environmental heavy metals-induced neurotoxicity and Alzheimer’s-like disease in rats. *Food Chem. Toxicol.* **111**, 432–444 (2018). 29170048 10.1016/j.fct.2017.11.025

[20] Wu, X. H. et al. Reversal of hyperglycemia in diabetic rats by portal vein transplantation of islet-like cells generated from bone marrow mesenchymal stem cells. *World J. Gastroenterol.* **13**, 3342–3349 (2007). 17659673 10.3748/wjg.v13.i24.3342 PMC4172714

[21] McCormick, D. L. et al. Overexpression of Cyclooxygenase-2 in rat oral cancers and prevention of oral carcinogenesis in rats by selective and nonselective COX inhibitors. *Cancer Prev. Res.* **3**, 73–81 (2010). 10.1158/1940-6207.CAPR-09-0151 PMC2804934 20051374

[22] Saini, U. et al. Preconditioning mesenchymal stem cells with caspase Inhibition and hyperoxia prior to hypoxia exposure increases cell proliferation. *J. Cell. Biochem.* **114**, 2612–2623 (2013). 23794477 10.1002/jcb.24609 PMC4017598

[23] Suzuki, Y. et al. Effects of prolonged water washing of tissue samples fixed in formalin on histological staining. *Biotech. Histochem.* **87**, 241–248 (2012). 21958122 10.3109/10520295.2011.613410 PMC3793282

[24] Sabine, L. & Brian, S. Everitt. in *A Handbook of Statistical Analyses Using SPSS* 145–149 (Chapman & Hall/CRC, (2004).

[25] Sherman, A. B., Gilger, B. C., Berglund, A. K. & Schnabel, L. V. Effect of bone marrow-derived mesenchymal stem cells and stem cell supernatant on equine corneal wound healing in vitro. *Stem Cell. Res. Ther.* **8**, 1–10 (2017). 28545510 10.1186/s13287-017-0577-3 PMC5445363

[26] Kannan, S., Gokul Krishna, S., Gupta, P. K. & Kolkundkar, U. K. Advantages of pooling of human bone marrow-derived mesenchymal stromal cells from different donors versus single-donor MSCs. *Sci. Rep.* **14**, 1–13 (2024). 10.1038/s41598-024-62544-8 PMC11144708 38825595

[27] Luisi, J. et al. Concentration-associated pathology of alkali burn in a mouse model using anterior segment optical coherence tomography with angiography. *Exp. Eye Res.* **223**, 109210 (2022). 35987418 10.1016/j.exer.2022.109210 PMC12570154

[28] Mokhber Dezfouli, M. R. et al. Intrapulmonary autologous transplant of bone marrow-derived mesenchymal stromal cells improves lipopolysaccharide-induced acute respiratory distress syndrome in rabbit. *Crit. Care*. **22**, 1–13 (2018). 30572913 10.1186/s13054-018-2272-x PMC6302408

[29] Cade, F. et al. Alkali burn to the eye: protection using TNF-α Inhibition. *Cornea* **33**, 382–389 (2014). 24488127 10.1097/ICO.0000000000000071

[30] Santacruz, C. et al. Expression of IL-8, IL-6 and IL-1β in tears as a main characteristic of the immune response in human microbial keratitis. *Int. J. Mol. Sci.* **16**, 4850–4864 (2015). 10.3390/ijms16034850 PMC4394453 25741769

[31] Liang, W. et al. Zeb1 regulation of wound-healing-induced inflammation in alkali-damaged Corneas. *iScience* **25**, 104038 (2022). 35340433 10.1016/j.isci.2022.104038 PMC8941209

[32] Osawa, Y. et al. Tumor necrosis factor alpha-induced interleukin-8 production via NF-κB and phosphatidylinositol 3-kinase/Akt pathways inhibits cell apoptosis in human hepatocytes. *Infect. Immun.* **70**, 6294–6301 (2002). 12379708 10.1128/IAI.70.11.6294-6301.2002 PMC130316

[33] Tao, H. et al. Mesenchymal stem cell-derived extracellular vesicles for corneal wound repair. *Stem Cells Int.* **2019**, 5738510 (2019). 10.1155/2019/5738510 PMC6925772 31885617

[34] Zhou, C. & Bai, X. Y. Strategies for the induction of anti-inflammatory mesenchymal stem cells and their application in the treatment of immune-related nephropathy. *Front. Med. (Lausanne)*. **9**, 891065 (2022). 36059816 10.3389/fmed.2022.891065 PMC9437354

[35] Hakami, N. Y., Dusting, G. J., Chan, E. C., Shah, M. H. & Peshavariya, H. M. Wound healing after alkali burn injury of the cornea involves Nox4-Type NADPH oxidase. *Invest. Ophthalmol. Vis. Sci.* **61**, 20–20 (2020). 33079994 10.1167/iovs.61.12.20 PMC7585390

[36] Oh, J. Y. et al. The Anti-Inflammatory and Anti-Angiogenic role of mesenchymal stem cells in corneal wound healing following chemical injury. *Stem Cells*. **26**, 1047–1055 (2008). 18192235 10.1634/stemcells.2007-0737

[37] Lan, W., Petznick, A., Heryati, S., Rifada, M. & Tong, L. Nuclear Factor-κB: central regulator in ocular surface inflammation and diseases. *Ocul Surf.* **10**, 137–148 (2012). 22814642 10.1016/j.jtos.2012.04.001

[38] Noort, A. R. et al. NF-κB-inducing kinase is a key regulator of inflammation-induced and tumour-associated angiogenesis. *J. Pathol.* **234**, 375–385 (2014). 25043127 10.1002/path.4403 PMC4194146

[39] Van Hinsbergh, V. W. M. & Koolwijk, P. Endothelial sprouting and angiogenesis: matrix metalloproteinases in the lead. *Cardiovasc. Res.* **78**, 203–212 (2008). 18079100 10.1093/cvr/cvm102

[40] Liu, S. et al. Gene-based antiangiogenic applications for corneal neovascularization. *Surv. Ophthalmol.* **63**, 193–213 (2018). 29080632 10.1016/j.survophthal.2017.10.006

[41] Kvanta, A., Sarman, S., Fagerholm, P., Seregard, S. & Steen, B. Expression of matrix Metalloproteinase-2 (MMP-2) and vascular endothelial growth factor (VEGF) in Inflammation-associated corneal neovascularization. *Exp. Eye Res.* **70**, 419–428 (2000). 10865990 10.1006/exer.1999.0790

[42] Zhang, J., Wang, S., He, Y., Yao, B. & Zhang, Y. Regulation of matrix metalloproteinases 2 and 9 in corneal neovascularization. *Chem. Biol. Drug Des.* **95**, 485–492 (2020). 31002472 10.1111/cbdd.13529

[43] Ma, Y. et al. Reconstruction of chemically burned rat corneal surface by bone Marrow–Derived human mesenchymal stem cells. *Stem Cells* **24**, 315–321 (2006). 16109757 10.1634/stemcells.2005-0046

[44] Salari, V., Mengoni, F., Gallo, F., Del, Bertini, G. & Fabene, P. F. The anti-inflammatory properties of mesenchymal stem cells in epilepsy: possible treatments and future perspectives. *Int J. Mol. Sci.* **21**, 9683 (2020). 10.3390/ijms21249683 PMC7765947 33353235

[45] Li, F. & Zhao, S. Z. Mesenchymal stem cells: potential role in corneal wound repair and transplantation. *World J. Stem Cells*. **6**, 296–304 (2014). 25126379 10.4252/wjsc.v6.i3.296 PMC4131271

[46] Gu, X. J. et al. Involvement of NADPH oxidases in alkali burn-induced corneal injury. *Int. J. Mol. Med.* **38**, 75–82 (2016). 27221536 10.3892/ijmm.2016.2594 PMC4899027

[47] Sahreen, A., Fatima, K., Zainab, T. & Saifullah, M. K. Changes in the level of oxidative stress markers in Indian catfish (Wallago attu) infected with isoparorchis Hypselobagri. *Beni Suef Univ. J. Basic. Appl. Sci.* **10**, 1–8 (2021).

[48] Assady, M., Farahnak, A., Golestani, A. & Esharghian, M. R. Superoxide dismutase (SOD) enzyme activity assay in fasciola spp. Parasites and liver tissue extract. *Iran. J. Parasitol.* **6**, 17–22 (2011). 22347309 PMC3279904

[49] Jung, K. J. et al. Mesenchymal stem cells decrease oxidative stress in the bowels of Interleukin-10 knockout mice. *Gut Liver*. **14**, 100–107 (2020). 31158947 10.5009/gnl18438 PMC6974321

[50] Yang, R. L. et al. Antioxidant mechanisms of mesenchymal stem cells and their therapeutic potential in vitiligo. *Front. Cell. Dev. Biol.* **11**, 1293101 (2023). 38178870 10.3389/fcell.2023.1293101 PMC10764575

[51] Tobita, Y. et al. Effects of selective peroxisome proliferator activated receptor agonists on corneal epithelial wound healing. *Pharmaceuticals* **14**, 88 (2021). 33504094 10.3390/ph14020088 PMC7911852

[52] Wu, Y. & Zhou, B. P. TNF-α/NF-κB/Snail pathway in cancer cell migration and invasion. *Br. J. Cancer 2010*. **102**, 639–644 (2010). 10.1038/sj.bjc.6605530 PMC2837572 20087353

[53] Prabahar, A. et al. Unraveling the complex relationship between mRNA and protein abundances: a machine learning-based approach for imputing protein levels from RNA-seq data. *NAR Genom. Bioinform.* **6**, 14523 (2024). 10.1093/nargab/lqae019 PMC10858678 38344273

[54] Kim, D. W. et al. PEP-1-FK506BP inhibits alkali burn-induced corneal inflammation on the rat model of corneal alkali injury. *BMB Rep.* **48**, 618–623 (2015). 25817214 10.5483/BMBRep.2015.48.11.041 PMC4911203

[55] Renner, F. & Schmitz, M. L. Autoregulatory feedback loops terminating the NF-κB response. *Trends Biochem. Sci.* **34**, 128–135 (2009). 19233657 10.1016/j.tibs.2008.12.003

[56] Rothschild, D. E., McDaniel, D. K., Ringel-Scaia, V. M. & Allen, I. C. Modulating inflammation through the negative regulation of NF-κB signaling. *J. Leukoc. Biol.* **103**, 1131–1150 (2018). 10.1002/JLB.3MIR0817-346RRR PMC6135699 29389019

[57] Mao, H., Zhao, X. & Sun, S. C. NF-κB in inflammation and cancer. *Cell. Mol. Immunol.* **22**, 811–839 (2025). 10.1038/s41423-025-01310-w PMC12310982 40562870

[58] Hoesel, B. & Schmid, J. A. The complexity of NF-κB signaling in inflammation and cancer. *Mol. Cancer* **12**, 1–15 (2013). 10.1186/1476-4598-12-86 PMC3750319 23915189

[59] Sayed, A. H., Mahmoud, N. S., Mohawed, O. A. M. & Ahmed, H. H. Combined effect of Pantoprazole and mesenchymal stem cells on experimentally induced gastric ulcer: implication of oxidative stress, inflammation and apoptosis pathways. *Inflammopharmacology* **32**, 1961–1982 (2024). 38652367 10.1007/s10787-024-01469-0 PMC11136780

[60] Yang, C. M. et al. Upregulation of COX-2 and PGE2 induced by TNF-α mediated through TNFR1/MitoROS/PKCα/P38 MAPK, JNK1/2/FoxO1 cascade in human cardiac fibroblasts. *J. Inflamm. Res.* **14**, 2807 (2021). 34234507 10.2147/JIR.S313665 PMC8254141

[61] Medina, J. P. et al. MSC therapy ameliorates experimental gouty arthritis hinting an early COX-2 induction. *Front. Immunol.* **14**, 1193179 (2023). 37533852 10.3389/fimmu.2023.1193179 PMC10391650

[62] Turner, M. D., Nedjai, B., Hurst, T. & Pennington, D. J. Cytokines and chemokines: at the crossroads of cell signalling and inflammatory disease. *Biochim. Biophys. Acta (BBA) - Mol. Cell. Res.* **1843**, 2563–2582 (2014). 10.1016/j.bbamcr.2014.05.014 24892271

[63] Wu, J. et al. TNF-α contributes to sarcopenia through caspase-8/caspase-3/GSDME-mediated pyroptosis. *Cell Death Discov.* **9**, 1–15 (2023). 10.1038/s41420-023-01365-6 PMC9950087 36823174

[64] Zhang, L. et al. Mesenchymal stem cells alleviate acute lung injury and inflammatory responses induced by Paraquat poisoning. *Med. Sci. Monit.* **25**, 2623–2632 (2019). 30967525 10.12659/MSM.915804 PMC6474293

[65] Slama, Y. et al. The dual role of mesenchymal stem cells in cancer pathophysiology: pro-Tumorigenic effects versus therapeutic potential. *Int. J. Mol. Sci.*. **24**, 13511 (2023). 37686315 10.3390/ijms241713511 PMC10488262

[66] Viveiros, M. M. H., Zucoloto, L. H., Shiguematsu, Á. I., Rainho, C. A. & Schellini, S. A. Comparison of techniques for corneal epithelium cell culture for the collection of conditioned medium. *Arq. Bras. Oftalmol* **87**, 1425 (2024). 10.5935/0004-2749.2022-0084 PMC11619711 38655938

[67] Pellegrini, G. et al. p63 identifies keratinocyte stem cells. *Proc. Natl. Acad. Sci. U S A*. **98**, 3156–3161 (2001). 11248048 10.1073/pnas.061032098 PMC30623

[68] Rohaina, C. M. et al. Reconstruction of limbal stem cell deficient corneal surface with induced human bone marrow mesenchymal stem cells on amniotic membrane. *Transl. Res.* **163**, 200–210 (2014). 24286920 10.1016/j.trsl.2013.11.004

