# Supplementary figures and images for: The efficacy of bone marrow-derived mesenchymal stem cells in restoring limbal stem cell deficiency in rat model
Source: Sci Rep. 2025 Nov 20;15:41168. doi: 10.1038/s41598-025-26637-2 (PMC12638304; doi:10.1038/s41598-025-26637-2)

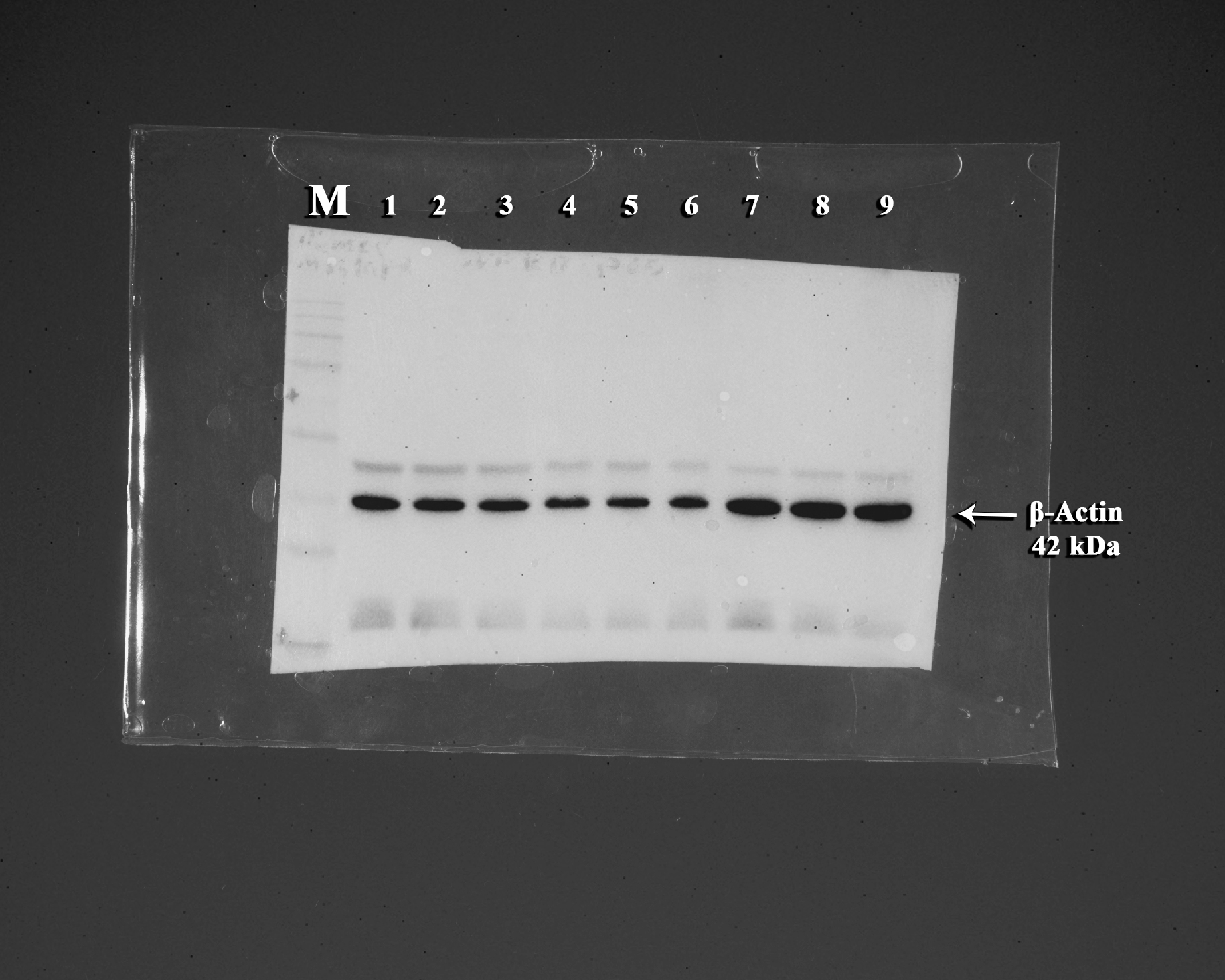

Supplement: Supplementary file 1 — Supplementary Material 1 [file 41598_2025_26637_MOESM1_ESM.jpg]

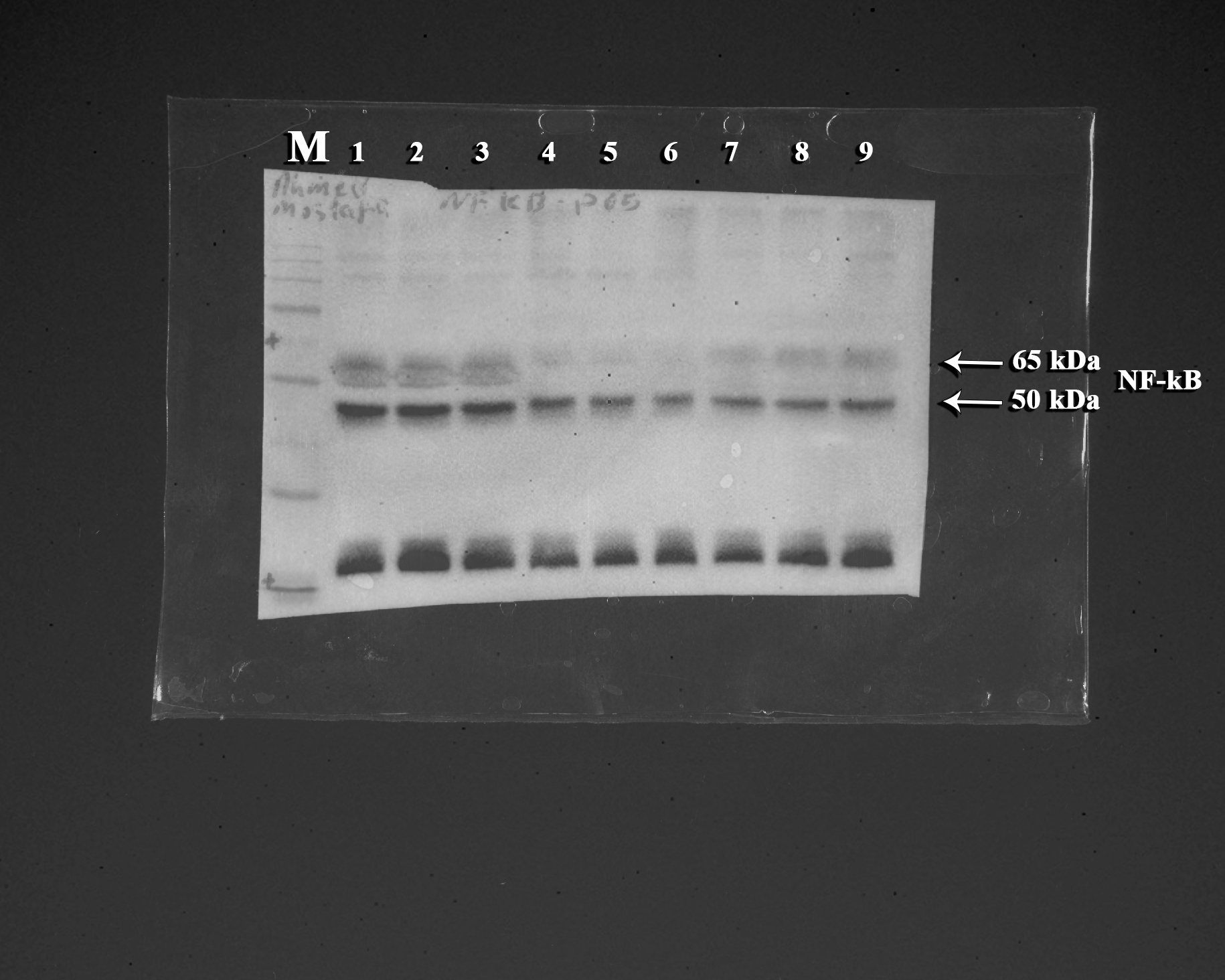

Supplement: Supplementary file 2 — Supplementary Material 2 [file 41598_2025_26637_MOESM2_ESM.jpg]
