# Supplementary material for: The efficacy of bone marrow-derived mesenchymal stem cells in restoring limbal stem cell deficiency in rat model
Source: Sci Rep. 2025 Nov 20;15:41168. doi: 10.1038/s41598-025-26637-2 (PMC12638304; doi:10.1038/s41598-025-26637-2)
Supplement: Supplementary file 3 — Supplementary Material 3 [file 41598_2025_26637_MOESM3_ESM.docx]

**The efficacy of bone marrow-derived mesenchymal stem cells in restoring limbal stem cell deficiency in rat model**

**Mohamed A. El-Desouky ^1^, Nadia S. Mahmoud ^2,3^, Fayek M. Ghaleb ^4^,** **Fatma B. Rashidi ^1^, Iman M.A.Zaki ^5^, Marwa A. Fouly ^6^, Ahmed M. Ata ^7*^, Hanaa H. Ahmed ^2,3^**

^1^ Biochemistry Division, Chemistry Department, Faculty of Science, Cairo University, Giza, Egypt

^2^ Hormones Department, Medical Research and Clinical Studies Institute, National Research Centre, Dokki, Giza, Egypt

^3^ Stem Cells Lab, Center of Excellence for Advanced Sciences, National Research Centre, Dokki, Giza, Egypt

^4^ Clinical Pathology Department, Research Institute of Ophthalmology, Giza, Egypt.

^5^ Pathology Department, Research Institute of Ophthalmology, Giza, Egypt.

^6^ Ophthalmology Department, Research Institute of Ophthalmology, Giza, Egypt.

^7^ Biochemistry Department, Research Institute of Ophthalmology, Giza, Egypt.

***Corresponding Author: Ahmed M. Ata**

**Email:** [**ahmed.mostafa@rio.sci.eg**](mailto:ahmed.mostafa@rio.sci.eg)**, ORCID: 0000-0003-1969-9707**

**Emails and ORCID of authors:**

Prof. Dr. Mohamed A El-Desouky, E-mail: meldesouky@sci.cu.edu.eg, ORCID: 0000-0001-5007-5455

Dr. Nadia Samy Mahmoud, E-mail: [nadiasamy@sci.asu.edu.eg](mailto:nadiasamy@sci.asu.edu.eg) , ORCID: 0000-0002-9085-4063.

Prof. Dr. Fayek M. Ghaleb, E-mail: fayek.ghaleb@gmail.com

Ass. Prof. Fatma B. Rashidi, Email: fabdallah@sci.cu.edu.eg, ORCID: 0000-0003-1646-0856

Prof. Dr**.** Iman M.A.Zaki, Email: imanmz45@gmail.com

Ass. Prof. Marwa A. Fouly, Email: marwa.rio2014@gmail.com

Prof. Dr. Hanaa Hamdy Ahmed, E-mail: [hh.ahmed@nrc.sci.eg](mailto:hh.ahmed@nrc.sci.eg), ORCID: 0000-0001-8642-9251


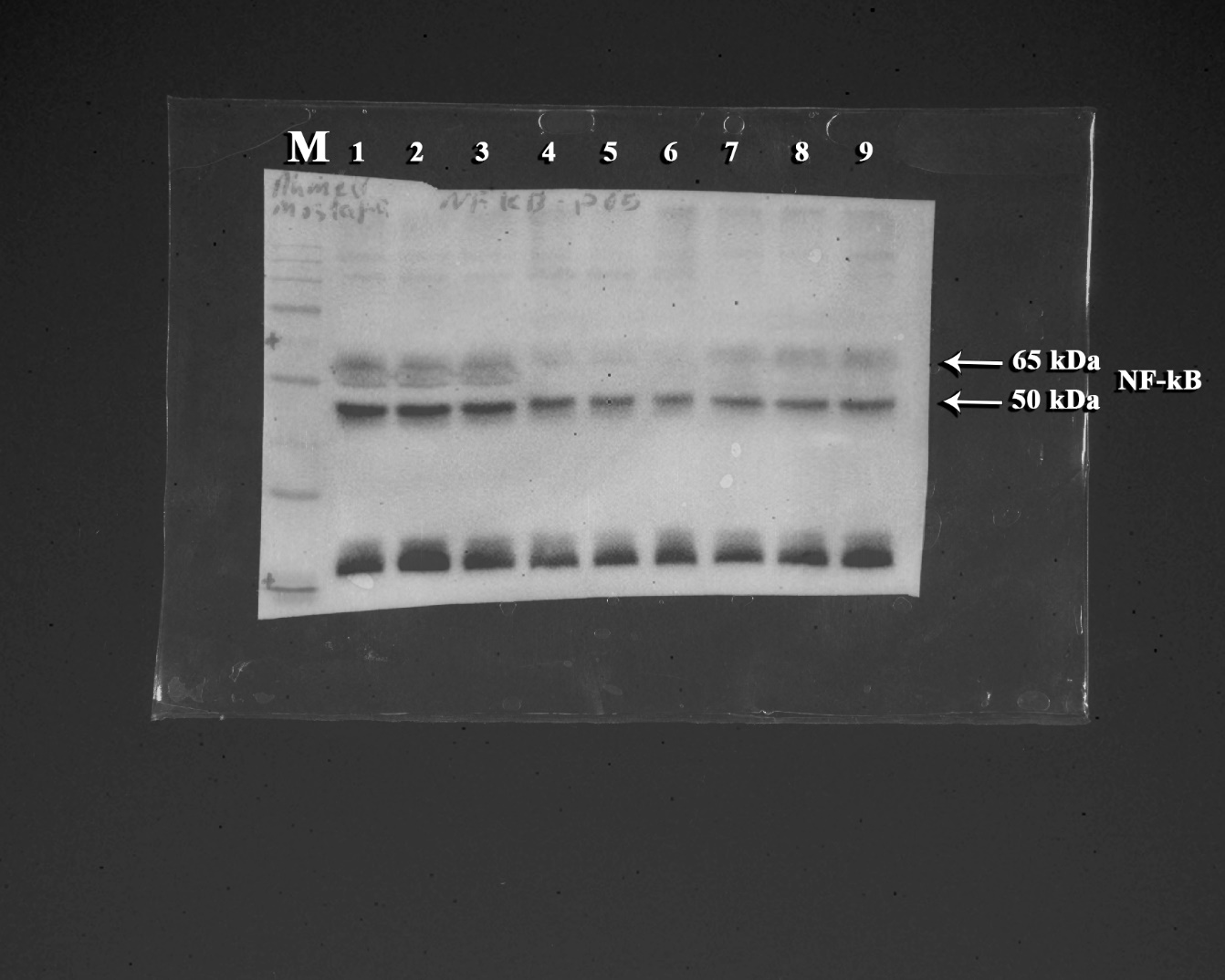


**Figure S1**: Original uncropped blot of protein expression patterns of NF-kB in the different studied groups. M: Molecular weight marker, bands (1-3): control group, bands (4-6): stem cells group, bands (7-9): alkali burn group


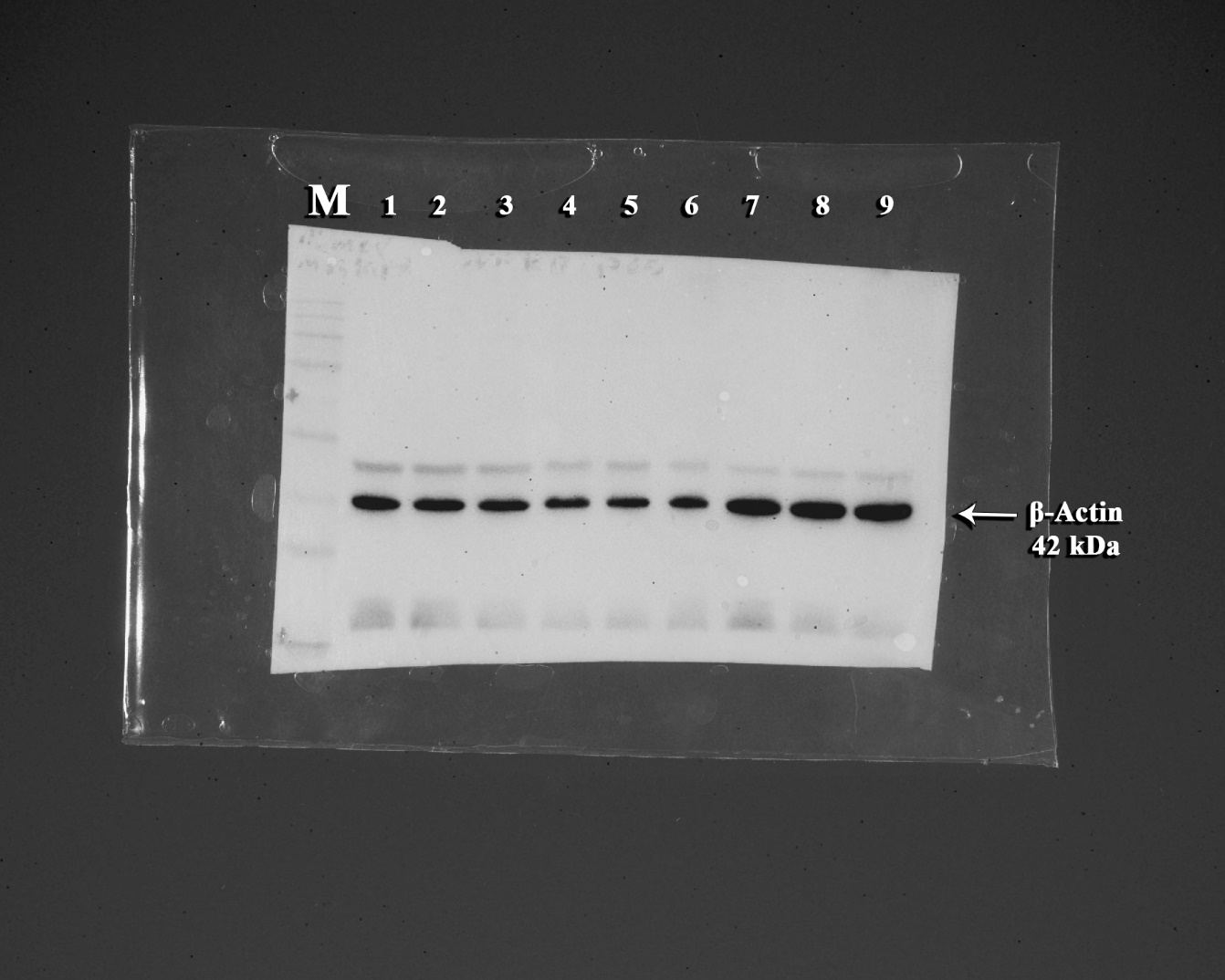


**Figure S2**: Original uncropped blot of protein expression patterns of β-actin in the different studied groups. M: Molecular weight marker, bands (1-3): control group, bands (4-6): stem cells group, bands (7-9): alkali burn group
